# First experimental evidence for olfactory species discrimination in two nocturnal primate species (*Microcebus lehilahytsara* and *M. murinus*)

**DOI:** 10.1038/s41598-019-56893-y

**Published:** 2019-12-31

**Authors:** Annika Kollikowski, Elke Zimmermann, Ute Radespiel

**Affiliations:** Institute of Zoology, University of Veterinary Medicine Hannover, Hannover, Germany

**Keywords:** Speciation, Animal behaviour

## Abstract

Olfactory communication is highly important for nocturnal mammals, especially for solitary foragers, but knowledge is still limited for nocturnal primates. Mouse lemurs (*Microcebus* spp.) are nocturnal solitary foragers with a dispersed lifestyle and frequently use chemo-sensory signalling behaviour for governing social interactions. Different mouse lemur species can co-occur in a given forest but it is unknown whether olfaction is involved in species recognition. We first screened 24 captive mouse lemurs (9 *M. murinus*, 15 *M. lehilahytsara*) for their olfactory learning potential in an experimental arena and then tested the species discrimination ability with urine odour in an operant conditioning paradigm in four individuals. The majority of the screened animals (75%) did not pass the screening criteria within a 2-week test period. However, all four final test animals, two *M. murinus* and two *M. lehilahytsara*, were successfully trained in a 5-step-conditioning process to reliably discriminate conspecific from heterospecific urine odour (requiring an overall median of 293 trials). Findings complement previous studies on the role of acoustic signalling and suggest that olfaction may be an important additional mechanism for species discrimination.

## Introduction

The olfactory sense is of high significance for many mammals. It is used to recognize specific odours to avoid predators^1,2^, locate suitable food sources^3–7^, recognize kin^8–10^, or to find potential mates^9,11^ and avoid inbreeding^12–15^. It is generally assumed that not all species rely equally on olfaction but that high olfactory sensitivity is most beneficial under certain socio-ecological conditions, such as nocturnality, solitary lifestyle, territoriality, or sympatry in cryptic species. For a long time, rodents and in particular lab mice and rats have been regarded as perfect models to study olfactory discrimination abilities and olfactory communication^16–19^. Their high olfactory sensitivity is based on a high number of olfactory receptors in the nose and an extraordinarily rich repertoire of vomeronasal receptors (V1R and V2R) in the vomeronasal organ (VNO)^20,21^. Such complex cognitive functions were interpreted as adaptive^22,23^ given for example the dispersed spatial organization of rodents.

The importance of olfactory communication in primates including humans, however, was long underestimated due to the assumption that they are microsmatic because of their predominantly diurnal and gregarious lifestyle, a largely reduced or even lacking VNO^24–26^ and few olfactory receptors^27^. Their olfactory abilities have only gained more scientific attention over the last two decades^24,26,28–30^. In haplorrhine primates, for example, mandrills of both sexes were observed to show flehmen behaviour in response to the presentation of conspecific odorants of males and females^31^. Furthermore, it was shown that rhesus macaques and chimpanzees could recognize group membership via olfactory cues^8,32^. Humans were described to use olfaction in kin recognition^33,34^ and mate choice^35^. Among new world primates, male pygmy marmosets showed an increased rate of mounting and piloerection as well as sniffing and licking of anogenital scents of females in the peri-ovulatory period compared with the non-ovulatory period^36^, and tufted capuchins are able to discriminate between the urine odours of three new world monkey species including their own^37^.

In strepsirrhine primates, most studies focused on the ring-tailed lemur (*Lemur catta*), a diurnal and group-living primate, endemic to Madagascar. This species produces scent by means of specialised scent glands on the forearms (brachial and antebrachial organs), scrotum, and perianal region^38^. It has been shown that *L. catta* recognize conspecific individuals by scent^39^, that male scent advertises genetic quality and relatedness^40,41^ and that injuries can be detected via scent^42^.

However, a study of delBarco-Trillo *et al*.^43^ showed that those strepsirrhine species which mainly mark with urine, have a greater chemical complexity and are more distinct from each other in their urine than those marking mainly via glandular secretions like the ring-tailed lemur^43^. The authors suggested that these species evolved species-specific urinary signatures due to their nocturnal and solitary lifestyle^43^. A recent study on the protein content of mouse lemur urine revealed a new urinary protein, WFDC12, that differs between *Microcebus murinus* and *M. lehilahytsara* by one amino acid^44^. Unfortunately, it is still not known if and how this protein is used in the olfactory communication of mouse lemurs and why it was only found in very high quantities in the urine of some, but not all males and not in female urine samples^44^. In order to test the hypothesis of species-specific urinary signatures, it is necessary to demonstrate that nocturnal primate species can discriminate between conspecific and heterospecific urine.

The nocturnal mouse lemurs (*Microcebus* spp.) with currently 24 described species inhabit the forest habitats of Madagascar, as reviewed in Zimmermann and Radespiel^45^. They are arboreal, solitary foragers and live in dispersed but individualized neighbourhoods^46^. Within these networks, female mouse lemurs often form stable, in most cases matrilinear sleeping groups, whereas males may or may not form sleeping groups, depending on the respective species^47–53^. To coordinate group members for their reunion at the end of the night, mouse lemur gathering calls carry group-specific acoustic signatures^54^. In addition, vocalizations do contain individual signatures^55,56^.

It is known that up to two species of mouse lemurs can co-occur in a given forest and most of these cases of sympatry concern the widely distributed grey mouse lemur (*M. murinus*) that can overlap with a more local or regional congener in the western half of Madagascar^57^. However, hybrids were so far only observed between *M. murinus* and *M. griseorufus*^58–60^, leading to the assumption that mouse lemurs should have a species recognition system. It was already shown that mouse lemurs react differently to calls from mouse lemur males of different species and that conspecific calls emit the highest reaction^56^, but it is not known if olfactory cues are also used for species discrimination.

Mouse lemurs have a well-developed rhinarium and probably the largest repertoire of functional VNO receptor genes among primates^61–64^ and therefore are a promising model to study their olfactory abilities. They mark via urine (deposited during urine washing, rhythmic micturition or anogenital rubbing) but also via other secretions (saliva, glandular) deposited during mouth wiping or anogenital rubbing^65^. Olfactory marking behaviour was described in many different contexts, leading to the assumption that much more information is conveyed via olfaction than is known to date. For example, sleeping sites are marked when the animals leave their shelter at the beginning of the night^54^, intraspecific dominance between males was shown to be communicated via urine^66,67^, and females show marking behaviour more frequently during than outside oestrus to advertise their reproductive state to possible mates^68^. Whereas male mouse lemurs typically compete when localizing receptive females during the short reproductive season^69–71^, females can also refuse to mate with certain males due to a lack of sexual dimorphism and due to female dominance, at least in some species^72–78^.

Considering this socio-ecological background, selection should favour the evolution of a reliable species recognition mechanism in both sexes that would allow to save energy or injury costs during the search for a suitable mate and to avoid hybridization with a sympatric congener. Given the need for quick and reliable decisions to be taken by potential mouse lemur mates of both sexes within the mating context, it would be beneficial to complement the acoustic species recognition system with olfactory species recognition to increase the reliability of the communication system. This could be advantageous, since animals can perceive chemical signals even when the sender is no longer at the same site and therefore can use them as a trace to find possible interaction partners. Furthermore, producing one longer lasting chemical signal of presence should be less costly in terms of energy, loss of feeding time and potential predator attraction, than to vocalize frequently or constantly which would be needed to serve the same purpose.

The aim of this project is therefore to investigate the sensory olfactory capabilities of two nocturnal primates (*Microcebus* spp.) in the context of species discrimination. For this purpose, a new operant conditioning paradigm was developed and we further evaluate the general suitability of this setup for training mouse lemurs and hypothesize that mouse lemurs can be trained via operant conditioning to discriminate between a conspecific and a heterospecific urine odour.

## Results

### Pilot phase and initial screening

**Figure 1:**
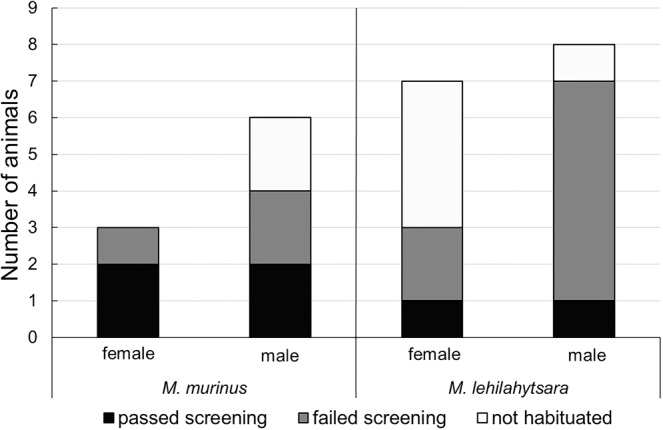
Number of suitable versus excluded test animals in pilot phase and screening. Animals that fulfilled all screening criteria are marked as “passed screening”, animals that were excluded during TS1a are marked as “failed screening” and animals that could not be habituated are marked as “not habituated”.

After the pilot phase and the initial screening, only 6 out of 24 animals (25%) have passed the screening criteria and were considered suitable for the operant conditioning process (as shown in Fig. 1 and in Supplementary Table S2). These six animals consisted of two *M. lehilahytsara* (1 m + 1 f) and four *M. murinus* (2 m + 2 f). Five of the suitable animals were aged 2–3 years, whereas the sixth animal was six years old. In contrast, the other 18 animals (5 *M. murinus*, 13 *M. lehilahytsara*, aged one to eight years) did not achieve habituation (n = 7) within three test days or did not fulfil the screening criteria within 10 test days (n = 11) and were therefore not considered suitable for further training (Fig. 1). The overall success rate during habituation and initial screening was more than three times higher in *M. murinus* (44.4%) than in *M. lehilahytsara* (13.3%) but did not differ substantially between males (21.4%) and females (30%, Fig. 1). Animals, which passed the habituation but not the screening period, were excluded from further training due to frantic movements and/or freezing behaviour or to a secondary decrease in the number of conducted trials/day to <10 as well as missing sniffing behaviour (see Supplementary Table S2).

### Overall time needed for conditioning process

The training throughout all five learning steps of the four test animals to discriminate conspecific urine odour from heterospecific urine odour took the overall median of 27.5 test days and 293 trials (Tables 1, 2 and 3). The overall length of the learning period differed between the four animals from 20–31 days and between 243–346 trials. Whereas the two *M. lehilahytsara* (*M. l*., GND, GIN) learned faster (243, 250 trials), the two *M. murinus* (*M. m*.) needed more trials (336, 346 trials) but not necessarily more days in total to complete the learning steps (Table 1). GND was part of a pilot phase three months prior to this study and therefore familiar with the general experimental procedure. He learned the quickest (20 days) and conducted 15 trials/day from the fifth experimental day onwards. However, he was not the quickest across all test series, but only in TS1a and in TS3 (Table 1).

**Table 1:** Time needed for operant conditioning (without initial screening/pilot phase).

Animal ID & species	Sex	Test Series										Total		Median Trials/Day
		TS1a		TS1b		TS1c		TS2		TS3				
		Days	Trials	Days	Trials	Days	Trials	Days	Trials	Days	Trials	Days	Trials	
GND (*M. l*.)	m	10	100	2	30	4	60	2	30	2	30	20	250	15
GIN (*M. l*.)	f	13	94	7	42	5	40	2	20	4	47	31	243	8
PUM (*M. m*.)	m	12	158	3	39	3	45	5	64	3	40	26	346	15
LIL (*M. m*.)	f	11	129	2	20	9	105	4	48	3	34	29	336	11
Median		11.5	114.5	2.5	34.5	4.5	52.5	3	39	3	37	27.5	293

**Table 2:** Discrimination of water vs. conspecific urine odour across the last 20 trials in TS2.

Animal ID	Correct trials	False trials	p-value (Binomial test)	Correct trials [%]
GND	19	1	<0.0001***	95
GIN	18	2	<0.001***	90
PUM	18	2	<0.001***	90
LIL	17	3	0.003**	85

**Table 3:** Discrimination of conspecific vs. heterospecific urine odour across the last 20 trials in TS3.

Animal ID	Correct trials	False trials	p-value (Binomial test)	Correct trials [%]
GND	18	2	<0.001 ***	90
GIN	17	3	0.003 **	85
PUM	18	2	<0.001 ***	90
LIL	15	5	0.04 *	75

### Discrimination of urine odour from water in TS2

After completing all operant conditioning steps with only one urine odour type presented in the setup (TS1c and TS2), all four animals reached the learning criterion of ≥80% of correct trials (=conspecific odour corridor chosen) across the last 20 trials. This result was also highly significant in all four animals (Table 2).

### Discrimination of conspecific versus heterospecific urine odour in TS3

After the training in TS3 (2–4 days over a total of 30–47 trials), the four test animals showed a significant discrimination of the conditioned conspecific urine odour from a heterospecific urine odour (Table 3). Three out of four animals also reached the criterion of successful learning (≥80% of correct trials).

### Olfactory learning by operant conditioning

**Figure 2:**
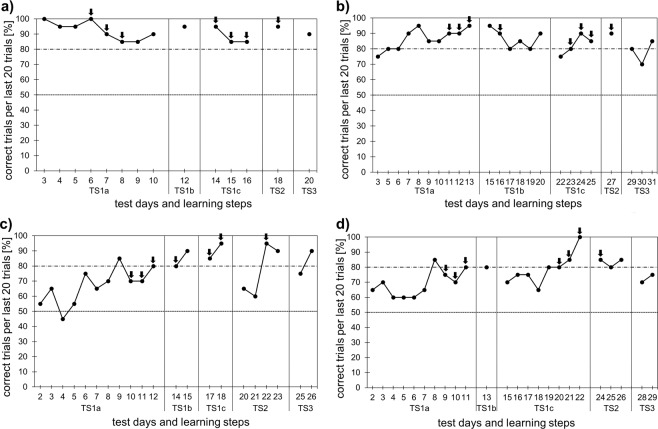
Learning curves of the four test animals (**a**) GND; (**b**) GIN; (**c**) PUM; (**d**) LIL across the five learning steps (Test Series 1a-3). For each day (besides the first day of each learning step), the percentage of correct trials across the last 20 trials is shown. All values above 80% (upper dotted line) indicate successful learning. The lower dotted line indicates chance level (50%). The black arrows point to those days, where the odour source was experimentally reduced. Learning steps: TS1a = without urine, inner + outer banana only on one side, step-wise reduction of outer banana to ¼ cup if animal shows significantly low error rate; TS1b = no outer banana and reduction of inner banana to ¼ slice; TS1c = introduction of rewarded urine sample and step-wise reduction of inner banana to 1/32 slice; TS2 = without any banana, step-wise reduction of pipetted urine to 5 µl; TS3 = simultaneous presentation of rewarded and non-rewarded urine.

The performance of GND was above 80% right from the beginning of the experiments and remained high across all learning steps (Fig. 2a), most likely due to the learning experience from the pilot phase (see GNDs learning curve during the pilot phase in Supplementary Fig. S1). All other naïve test animals (Fig. 2b–d) showed increasing learning performance over time and three out of four test animals reached the criterion of successful learning at the end of TS3. One animal, LIL, did not reach the 80% criterion at the end, but the learning curve was increasing in TS3 and pointing towards the criterion (Fig. 2d). Unfortunately, tests with this animal were stopped at that point, since it had already reached significance across the tests of the last two days (Binomial test for n = 21, p = 0.027). Drops in performance while staying in one learning step were mostly due to the stepwise reduction of the amount of the odour presented, be it banana or urine (see Fig. 2, the black arrows point at those days). This effect, however, was more visible in the early training steps than in the later training steps.

## Discussion

This study provides first empirical evidence for the discrimination of species-specific olfactory signatures in nocturnal primates. The established operant conditioning setup was feasible for conditioning mouse lemurs on the odour of a conspecific urine mix. However, we faced some unexpected methodological limitations, which shall be discussed first.

We had to exclude 18 of 24 (75%) animals from the subsequent final conditioning experiments. Several reasons may explain this relatively high dropout rate. First, animals may not have participated well in a spatial setup if they suffered from acute stress^79,80^. Test animals exhibiting frantic movements and/or freezing behaviour in our study (16 of 18 excluded animals, 89%) only rarely showed sniffing and responded in no coordinated way to the offered olfactory stimuli in form of the banana reward, indicating that stress may have affected the behaviour of a larger number of animals.

Second, other studies have demonstrated some degree of neophobia and cautiousness of mouse lemurs in the context of open field experiments^81–83^ and have attributed the different behavioural responses to different personality types^84–86^. They also reported about a freezing response in open field experiments^82^ similar to that seen in wild grey mouse lemurs as anti-predator behaviour^87^. In fact, test animals appeared to fall into two behavioural types: first, the more nervous type showing frantic movements and/or freezing behaviour and second, the more relaxed type that remained calm in the arena and showed directed orientation responses (sniffing) towards the stimuli. The overall success rate during screening of *M. murinus* (44.4%) was over three times higher than that of *M. lehilahytsara* (13.3%). These findings might suggest a species difference in neophobia, leading to different experimental performance. However, such species differences were not reported for mouse lemurs so far and this hypothesis therefore requires further testing.

Previous studies also reported an effect of age on behaviour and personality traits in mouse lemurs^86,88^. However, those studies showed contrasting results with boldness increasing with age in wild males in one study^86^, but with shorter box emergence latencies in younger as well as higher agitation scores in older captive animals^88^. In our study, five of eleven younger animals (1–3 years old, 45.5%), but only one of 13 older animals (4–8 years old, 7.7%) passed the screening. A study on grey mouse lemurs in a touchscreen-based cognitive task already showed an age-related impairment in older individuals, which needed more trials to complete a learning step and additionally showed a deficit in their cognitive flexibility^89^. Combined with higher agitation scores in older animals^88^, this could be the reason for finding most of the suitable animals in the group of younger adults. Further studies will be needed to clarify the relative contribution of stress, personality and age on the performance in new experimental setups.

Dropout rates are relatively rarely reported in cognition studies. However, one other learning study on captive mouse lemurs reported the dropout rates during the training (7 of 12 (58%) animals excluded^90^). Animals in that study were locked in a small cognitive test chamber and had to interact with a touchscreen^90^. Another study on wild mouse lemurs reported dropout rates in the initial familiarization to a maze (21 of 86 (24%) animals excluded, eight of which could be familiarized on a subsequent day)^81^. For future refinement of our experimental setup, it may be one solution to choose a smaller starting place to reduce animal exposure and anxiety before taking spatial decisions. Another study design would be to conduct other complementary olfactory experiments in the home cage, where the animals are in their familiar surroundings and might be calmer, but olfactory stimulation would be much less controlled under these conditions.

It could be argued that habituation to this new setup could eventually be reached if the animals would be trained for a longer period of time. However, two of three male *M. lehilahytsara* tested in the pilot phase could still not be trained to discriminate banana odour from water (TS1a), even though this test series was conducted for 22 and 30 days, respectively. This observation was the reason for developing the quick screening protocol used in this study. An initial screening across a series of ten test days can be concluded to be feasible to quickly establish mouse lemur olfactory learning potential across a larger number of animals in a relatively short time. We therefore recommend implementing such a procedure in future operant conditioning studies.

One test animal, GND, was already trained in a pilot phase three months before the start of the tests and therefore was not naïve to the setup and experimental procedure across all conditioning steps. The previous experience of this animal is clearly visible when comparing its high percentage of correct trials (>80%) from the very start of the experiments conducted in October and November to those from the experiments of the pilot phase in May and June 2016 (45%). Obviously, the underlying olfactory learning was already completed during the pilot phase and was memorized for at least three months. Odour memory was also described in other studies; for example in *Ateles geoffroyi*, where the performance of the tested animals was not effected by a 4-weeks break after the animals had learned to discriminate between two odour samples^91^.

Interestingly, the two *M. lehilahytsara* learned faster than the two *M. murinus* which needed more trials in total to complete the experiments. With regard to the higher dropout rate of *M. lehilahytsara* in the screening, this was unexpected. A recent field study compared the ecological generalist *M. murinus* with the specialist *M. berthae* in their innovation ability and concluded that the specialist species was faster in succeeding in a problem-solving task than the generalist species^92^. This could potentially also explain the observed species differences in our study, but this hypothesis will need to be tested in the future when comparative cognitive datasets become available.

To conclude, we showed that mouse lemurs passing the screening criteria could be trained via operant conditioning to discriminate conspecific urine samples from water and heterospecific urine samples by scent alone. All four test animals learned to use olfaction to find the food reward and could be conditioned to link the food reward to the scent of conspecific urine. The overall time needed for training the animals is comparable to previous studies on operant conditioning for olfactory discrimination in primates (*A. geoffroyi*^91^*, Saimiri sciureus*^93^). Consistent with the study of Joly *et al*.^89^, the oldest individual (six-year-old *M. murinus* male PUM) needed most trials to complete the experiments. However, this setup is not suitable to establish a spontaneous interest in or a preference for particular odours, in other words to study their biological relevance.

It is known that grey mouse lemur females advertise their oestrus not only with more frequent scent marking behaviour but also with oestrus advertisement calls^68^. In the dense forest habitats of Madagascar, auditory and olfactory cues have the highest potential to be perceived by conspecifics and potential mates^94^. However, to ensure that conspecific mates are attracted, females should not only advertise their oestrus, but also their species and their locality. This should be particularly beneficial under limited vision, which is characteristic for their microhabitats in the fine-branche niche^68,95^. Previous studies on the acoustic communication of mouse lemurs^54,94,96,97^ also found an individually distinct and species-specific advertisement call, which is uttered by sexually active males^55,56^. Combined with the ability of mouse lemurs to discriminate mouse lemur species by scent, as shown in this study, these findings suggest a multimodal (here chemo-acoustic) species recognition system in mouse lemurs.

*Multimodal* means, as defined by Slocombe, Walter and Liebal (2014), the “simultaneous combinations of signals from two or more modalities (gestural, facial, vocal and olfactory signals), and/or any signals requiring sensory integration by the receiver”^98^. Using multimodal cues for species recognition was already shown in other primate species (for example *Cebus apella*, visual-auditory matching^99^ and possible olfactory-visual matching in *Eulemur rufifrons*^100^). It is known that the capability to achieve a coherent and multimodal representation of the surroundings confers advantages, because it enables the receiver to achieve higher sensory resolution, which is the basis for well nuanced behavioural responses^101^. Additionally, multimodal cues are likely to “enhance the detection and discrimination of external stimuli”^101^ and increase the accuracy of decision making (e.g., in fish^102^).

To summarize, this study provides proof of principle for the discrimination of species-specific olfactory signatures by nocturnal primates. Findings are congruent with studies suggesting an important role of chemical signalling in species diversification^43,103^ which are most likely complemented by the use of auditory cues^54,56,94^ for species discrimination in mouse lemurs.

## Methods

### Ethical considerations

In this study we tested the animals in a non-invasive experimental setup. We performed all experiments according to the NRC Guide for the Care and Use of Laboratory Animals, the European Directive 2010/63/EU and the German Animal Protection law. The study was licensed by the Niedersächsisches Landesamt für Verbraucherschutz und Lebensmittelsicherheit (LAVES, reference number AZ 33.12-42502-04-14/1454) and followed the Principles for the Ethical Treatment of Non-human Primates of the American Society of Primatologists.

### Study animals and housing conditions

All study animals (except two *Microcebus lehilahytsara* who were born in Zoo Zurich, Switzerland) were born and kept individually or in groups of 2–3 animals of the same or opposite sex in the animal facility of the Institute of Zoology at the University of Veterinary Medicine, Hannover, Germany. All animals underwent routine weekly handling to check their health status and their reproductive state with standard procedures^68^. For detailed housing conditions see Hohenbrink *et al*.^73^. All cages were equipped with external mobile wooden nest boxes that were used to move the test animal from the home cage to the experimental room.

In a pilot phase during the breeding season 2016 (May – June), during which the steps for the operant conditioning (for details see below) were optimized, we tested three male *M. lehilahytsara* (age: 2–6 years), one of which (GND) completed all learning steps and two were trained in step TS1a only (for details see below).

In the transition period between reproductive and non-reproductive season (July – September 2016), we tested an additional 12 *M. lehilahytsara* (7 females (f), 5 males (m); age: 1–8 years) and 9 *M. murinus* (3 f, 6 m; age: 2–6 years) for their olfactory learning potential (for details see below) in an initial screening.

Due to the seasonality of mouse lemurs and daily time restrictions, only four animals (age: 2–6 years) subsequently underwent the complete test series of operant conditioning: two *M. lehilahytsara* (GIN (f), GND (m)) and two *M. murinus* (LIL (f), PUM (m)). That complete test series was conducted during the non-reproductive season in October - November 2016. One of these four test animals (GND) had already been trained in the pilot phase before (see above), where he completed all learning steps successfully (for learning curve see Supplementary Fig. S1) and therefore was not naïve when tested again three months later.

### Collection and preparation of urine samples

All urine samples of *M. murinus* and *M. lehilahytsara* had been collected during previous breeding seasons since 2014, but not during oestrus (in case of the females) to avoid potential biases from unknown hormonal status. Urine samples were collected in small inert glass vials (CS-Chromatographie Service GmbH, Langerwehe, Germany) during the weekly handling using single-use pipettes or in special nest boxes for urine collection with a grid as bottom, to which animals were confined prior to their active phase for one hour. Collected samples were directly frozen at −18 °C. To reduce the impact of individual scent signatures to a minimum, we mixed samples of three urine donors in equal parts at the beginning of a test series and froze them in aliquots of 5 µl and 18 µl at −18 °C until use. Sample donors were selected by two criteria: they have not been housed in the same room as the test animal over the last six months and were not first-degree relatives (r = 0.5) of the test animal.

### Experimental arena

**Figure 3:**
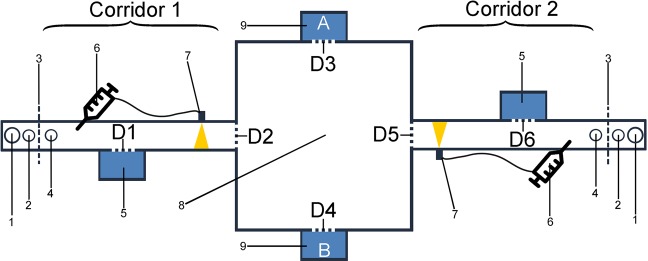
Experimental setup covered with acrylic glass. (1) non-transparent cup for “outer banana”; (2) transparent cup with filter paper for sample placement; (3) grid wall; (4) reward tray for “inner banana”; (5) nest box to enter at the end of each trial (transfer box); (6) apple juice reward injector connected to (7) light barrier; (8) central arena; (9) nest box on starting position (A/B = starting position); D1-6 = electronic doors accessible via remote control; size (LxWxH) of central arena = 70 cm × 70 cm × 40.5 cm; size of corridors = 70 cm × 8 cm × 9.5 cm.

The experiments were conducted in a separate experimental room which was divided into two parts, one with the experimental setup and one for experimenter monitoring. Due to the nocturnality of mouse lemurs, the experimental room was illuminated by dim red light during the experiments. The experimental setup (Fig. 3) consists of a central arena (Fig. 3: #8) that is connected to two 70 cm long corridors on opposing sides. The setup is topped by acrylic glass and the walls contained two more openings to attach nest boxes with the test animal to the setup (Fig. 3: #9). These served as start points for the experiments.

All four openings of the central arena are controlled via electronic doors (Fig. 3: D2–5), which can be opened and closed via remote control. At the front of the corridors is a light barrier installed (Fig. 3: #7), which is linked via cable to an injector (Fig. 3: #6) containing apple juice as reward that is ejected in portions of 80 µl at the far end of the corridor if the correct corridor has been chosen (for details about the learning steps see below). Next to the apple juice reward is a small dish (Fig. 3: #4) for the banana reward (=“inner banana”) in the first learning steps (for details see below). Each corridor ends with a plastic grid that prohibits exit but allows odour transmission (Fig. 3: #3). Behind the grid and therefore non-accessible for the animals are the conditioning stimuli. Closest to the grid is a small, transparent dish (Fig. 3: #2) on each side with filter paper (“Whatman^®^ glass microfiber filters, Grade GF/A, diameter 13 mm”, GE Healthcare, Solingen, Germany) for eventually presenting the urine odour and behind a bigger, non-transparent cup (Fig. 3: #1) that is used during the initial phases of the conditioning process and contains decreasing amounts of non-accessible banana (=“outer banana”). On the backside of each corridor is another electronic door (Fig. 3: D1 & D6) behind which a temporary nest box is placed (Fig. 3: #5). In these the animals can be moved back to the central arena after each test trial without further handling (=“transfer box”).

The experimental setup is surrounded by a thick curtain to visually separate the animal from the experimenter. The experimenter part of the room is equipped with a video monitor and a remote control unit to handle the electronic doors of the arena. Experiments were filmed from above with a camera (Sony Handycam DCR-SR210E) and simultaneously followed on the monitor behind the curtain. The following parameters were noted during each trial on a written protocol: beginning of the trial (time), end of the trial (time), chosen corridor (rewarded vs. non-rewarded) and behaviour in central arena (sniffing, freezing, frantic movements (for ethogram see Supplementary Table S1)).

### General experimental procedure

Each animal undergoing operant conditioning was eventually tested with two different urine mixes from the breeding season, one conspecific (Con) and one heterospecific (Het) urine mix. Each male was tested with a urine mix of three females and each female was tested with a urine mix of three males.

Prior to each experimental session and still during the sleep phase (light on), the test animal was locked into its home nest box for about half an hour. At the beginning of the animals’ active phase, when the animals are naturally hungry and motivated to forage for food, the test animal was carried to the experimental room in its home nest box and placed in the “A” or “B” position (Fig. 3A,B). The starting position (A or B) of the animal changed between each trial on one day. In addition, the starting position (A or B) changed in the first daily trial between consecutive test days. The corridor, which contained the rewarded conditioning stimulus changed daily too. A maximum of 15 trials was conducted per animal and day during a test session with a maximum duration of 1 hr.

Each trial started with opening the electronic door to the home next box (Fig. 3: D3/4) and the animal could enter the arena at its own will. The door was closed immediately when the animal had entered the arena. The animal could choose freely between the two corridors (Fig. 3: D2/5 open), but once it entered one of the corridors, the door between that corridor and the central arena was closed. If the animal did choose the corridor with the rewarded conditioning stimulus, it received 80 µl of apple juice and, if still at the beginning of the conditioning procedure, a small slice of banana at the end of the corridor (see below for learning steps). The door to the transfer box (Fig. 3: #5) next to the reward (Fig. 3: D1 or D6) was opened as soon as the animal took the reward and was closed immediately after the animal entered the transfer box. If the animal did choose the corridor without conditioning stimulus, it received no reward but had to wait for 30 sec, until the door (Fig. 3: D1 or D6) to the transfer box (Fig. 3: #5) was opened. This door was also closed after the animal entered the transfer box. The transfer box with the animal inside was moved to the new starting position A or B (Fig. 3) to start the next trial without further handling of the animal between trials. After each daily test session, the animal was brought back to its cage and the normal daily food was provided. Test animals were fed only after the test session to ensure a high foraging motivation during the experiments.

The arena and all external parts like tubes and small dishes etc. were wiped clean with hot water and 70% Ethanol and left air-drying after each test session. Furthermore, the door of the test room was left open and a fan was turned on between sessions to reduce the accumulation of animal, urine or reward smell as much as possible inside the experimental room.

### Pilot phase and initial screening for olfactory learning potential

All test animals were first habituated to the experimental setup. In the habituation phase, animals were encouraged to explore the arena and the corridors and to habituate to the artificial surrounding as well as the sound of the electronic doors. No urine odour, but only banana (odour) was presented in both non-transparent cups (Fig. 3: #1). The transparent cup for sample-placement remained empty (Fig. 3: #2). During habituation, the reward consisted of 1/8^th^ slice of banana (Fig. 3: #4) and about 80 µl apple juice, placed in both corridors. Frozen mealworms were also placed in the central arena and the corridors to encourage the animals to move around in the arena. Habituation was defined as being completed when the animal entered the arena voluntarily in <5 min and completed 10 trials/hr/day.

A pilot phase was conducted during the breeding season 2016 (May - July) with three *M. lehilahytsara* males to establish all experimental procedures and the succession of learning steps within the chosen paradigm. All animals were first habituated to the arena as described above. One animal, GND, also completed all learning steps afterwards (TS1a-TS3, for details see below). GND was also chosen for the subsequent period of data collection for this study and therefore was not naïve. The two other males, FIN and JUL, did not show successful learning in TS1a even after 22 and 30 test days, respectively, and did not enter any other learning step.

During a subsequent period of large-scale screening for their olfactory learning potential, 21 animals were tested on a maximum of 10 days in the setup. The aim of this screening was to quickly identify animals with a high olfactory learning potential for the subsequent operant conditioning, which needed to be completed before the end of the non-breeding season.

During the screening, animals underwent first a habituation phase (for details see above) and eventually moved on to the first learning step (TS1a) of the conditioning process (for details see below). Habituation was conducted for a maximum of three days and was defined as being completed, when the animal entered the arena voluntarily in <5 min and completed 10 trials/hr/day. If an animal could not be habituated within the given timeframe, it was excluded from further testing. The screening was conducted under the conditions of the first learning phase TS1a (see below) and started right after habituation. Animals were considered suitable for subsequent operant conditioning when they reached all of the following criteria within a maximum of seven days: (1) minimum of 10 trials/day possible within a 1 hr test session, (2) no frantic movements or longer freezing behaviour were shown in the arena, (3) sniffing behaviour (for ethogram see Supplementary Table S1) was shown. These criteria were chosen because a study on rhesus monkeys showed that the tendency to approach and sniff odour samples influenced the learning speed^104^ and the training of pigtailed macaques depended strongly on the motivation of the animals to sniff at an odour cue^105^. All parameters were noted on a written protocol. Due to daily time restrictions, four test animals were finally chosen for the subsequent operant conditioning.

### Learning steps of the conditioning procedure

The conditioning process consisted of five learning steps (Test Series (TS) 1a-c, TS2, TS3, for details see below), during which the animals had to learn to use their olfactory sense to obtain the reward. Animals moved from one learning step to the next when they showed significantly fewer errors than expected by chance (binomial distribution test, p < 0.05, expectation by chance = 0.5) across the last two test days. This criterion was used throughout the whole operant conditioning procedure. Apple juice was present in both injectors (Fig. 3: #6) throughout all learning steps, but the light barrier (Fig. 3: #7) did not activate the injector of the non-rewarded corridor. Hence, no apple juice was ejected when the animals entered the wrong corridor.

In TS1a, the conditioning odour (=“outer banana”, Fig. 3: #1) as well as the banana reward (=“inner banana”, Fig. 3: #4) were presented in one corridor only and only water was presented on the filter paper (Fig. 3: #2). The animal was rewarded with banana and apple juice. When the animal showed significantly fewer errors than expected by chance across the last two test days, the amount of the “outer banana” was stepwise reduced from a full cup to ¼ cup.

In TS1b, no “outer banana” was presented any more, but the amount of the “inner banana” was initially increased to ½ slice of banana to avoid a too fast decrease of banana odour. The amount of apple juice remained the same as in TS1a throughout the experiments of all learning steps. On the filter paper, water was presented only. When the animal showed significantly fewer errors than expected by chance across the last two test days, the amount of the “inner banana” was reduced to ¼ slice of banana.

In TS1c, the rewarded urine odour (Con) was introduced and presented together with small amounts of banana. For this, 10 µl of the urine mix were pipetted on the filter paper on one side, which were refreshed with 5 µl before each fourth trial. On the filter paper of the non-rewarded corridor, water was pipetted in the same amounts. When the animal showed significantly fewer errors than expected by chance across the last two test days, the amount of the inner banana was stepwise reduced from ¼ slice of banana to 1/32 slice.

From TS2 onwards, the animal was rewarded with apple juice only and no banana was present in the setup anymore. In TS2, the amount of the presented urine odour was reduced from 10 µl (refreshed with 5 µl) to 5 µl (refreshed with 2 µl) when the animal showed significantly fewer errors than expected by chance across the last two test days.

In the last step, TS3, the non-rewarded urine odour (Het) was introduced and presented instead of water behind the second corridor. In this step, the animals were trained to discriminate the rewarded conspecific urine odour and the non-rewarded heterospecific urine odour.

### Data analysis

Learning success was inspected after the end of the operant conditioning by calculating the percent of correct choices for a sliding window of the 20 last trials for each day during all phases of the operant conditioning to balance uneven trial numbers between days. For that, all trials of one day were filled up to 20 with the last trials from the day before. In accordance with other learning studies, 80% correct choices over the last 20 trials indicate successful learning^89^. The learning success was furthermore tested statistically by means of a Binomial test. When applied to the last 20 trials, ≥15 correct decisions (≥75% correct decisions) indicate a significant bias towards correct choices (p < 0.05).

The error rate was plotted in individual learning curves (for the raw data see Supplementary Tables S3–7) according to previous learning studies^89,90^. The first day of each new learning step was skipped (even the first two days of TS1a were skipped in GND and GIN due to low trial numbers) when plotting the learning curve and for the statistical analyses, because no 20 trials were available on Day 1 of each learning step due to the maximum of 15 trials/day. The fourth test day of GIN was not plotted, as the animal did not leave the starting box and therefore did not conduct any trial on this test day.

## Supplementary information

Supplementary Tables and Figure

## Data Availability

All data is included in this published article and its Supplementary Information File.
