# Supplementary material for: First experimental evidence for olfactory species discrimination in two nocturnal primate species (Microcebus lehilahytsara and M. murinus)
Source: Sci Rep. 2019 Dec 31;9:20386. doi: 10.1038/s41598-019-56893-y (PMC6938479; doi:10.1038/s41598-019-56893-y)
Supplement: Supplementary file 1 — Supplementary Tables and Figure [file 41598_2019_56893_MOESM1_ESM.pdf]

Title: First experimental evidence for olfactory species discrimination in two nocturnal primate species (*Microcebus lehilahytsara* and *M. murinus*)

Authors: Annika Kollikowski[1]*, Elke Zimmermann[1], Ute Radespiel[1]

[1]Institute of Zoology, University of Veterinary Medicine Hannover, Hannover, Germany

* Correspondence to be sent to: Annika Kollikowski, annika.kollikowski@tiho-hannover.de

Supplementary information

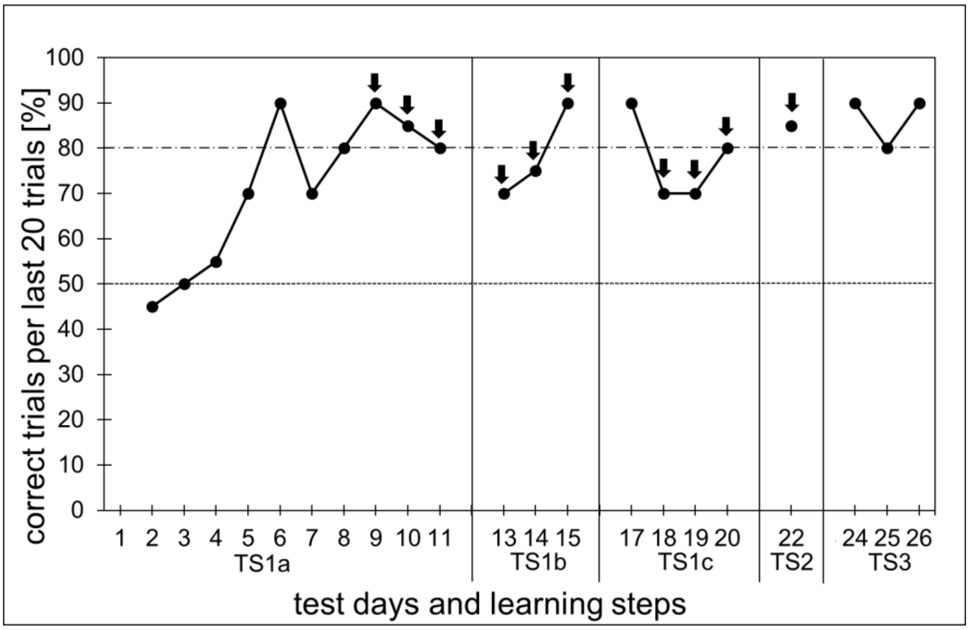

Figure S1: Learning curve of GND in the pilot phase. For each day (exclusive of the first day of each learning step), the percentage of correct trials across the last 20 trials is shown. All values above 80% (upper dotted line) indicate successful learning. The lower dotted line indicates chance level (50%). The arrows point on those days, where the odour source was experimentally reduced. Learning steps: TS1a = without urine, inner + outer banana only on one side, step-wise reduction of outer banana to ¼ cup if animal shows significantly low error rate; TS1b = no outer banana and reduction of inner banana to ¼ slice; TS1c = introduction of rewarded urine sample and step-wise reduction of inner banana to 1/32 slice; TS2 = without any banana, step-wise reduction of pipetted urine to 5µl; TS3 = simultaneous presentation of rewarded and non-rewarded urine.

Table S1: Ethogram used for categorizing the animals during the screening.

Behaviour	Description
Sniffing	the animal is standing still for a few seconds, sometimes bipedal, and pointing with the nose at one or both corridors alternatingly
Freezing	the animal is stationary, sometimes crouching, and not moving its body for > 10 s
Frantic movement	the animal runs very fast and without pause in the arena

Table S2: The behaviour of all test animals in pilot phase and screening. If a behaviour was shown at least once, it is marked with a "+". Behaviours that were never shown are marked with a "-".

	Animal ID	species	sex	age in 2016	considered suitable	conducted 10 trials/day	sniffing behaviour	frantic movement	freezing behaviour	completed habituation
Pilot phase	FIN	*M. lehihalytsara*	m	2	no	-	+	-	-	yes
	GND	*M. lehihalytsara*	m	3	yes	+	+	-	-	yes
	JUL	*M. lehihalytsara*	m	6	no	+	-	+	-	yes
Screening	ELI	*M. lehihalytsara*	f	1	no	-	-	-	+	no
	GIN	*M. lehihalytsara*	f	3	yes	+	+	-	-	yes
	HAP	*M. lehihalytsara*	f	4	no	-	-	+	-	no
	IMK	*M. lehihalytsara*	f	5	no	-	-	+	+	yes
	IRM	*M. lehihalytsara*	f	5	no	-	-	+	+	no
	LIA	*M. lehihalytsara*	f	8	no	-	-	+	+	no
	LIS	*M. lehihalytsara*	f	8	no	-	+	-	+	yes
	FEV	*M. lehihalytsara*	m	2	no	-	-	+	+	no
	FRO	*M. lehihalytsara*	m	2	no	+	+	+	+	yes
	GUD	*M. lehihalytsara*	m	3	no	-	-	+	-	yes
	LEO	*M. lehihalytsara*	m	8	no	+	+	+	-	yes
	LUP	*M. lehihalytsara*	m	8	no	-	-	-	-	yes
	LIL	*M. murinus*	f	2	yes	+	+	-	-	yes
	LOT	*M. murinus*	f	2	yes	+	+	-	-	yes
	NUR	*M. murinus*	f	4	no	-	-	+	+	yes
	LIM	*M. murinus*	m	2	no	+	-	+	-	yes
	MAN	*M. murinus*	m	3	yes	+	+	-	-	yes
	NAP	*M. murinus*	m	4	no	-	-	+	+	no
	NIG	*M. murinus*	m	4	no	-	-	+	-	no
	PRI	*M. murinus*	m	6	no	+	-	+	-	yes
	PUM	*M. murinus*	m	6	yes	+	+	-	-	yes
	= 24 animals tested in total *M. lehilahytsara* n = 15 *M. murinus* n = 9 aged 1-8 years				= 6 animals considered suitable	= 11 animals conducted 10 trials/each day	= 10 animals showed sniffing behaviour	= 14 animals showed frantic movement	= 9 animals showed freezing behaviour	= 17 animals completed habituation

Table S3: Raw data of GND in the pilot phase. A “c” stands for choosing the rewarded corridor (= correct). An “f” stands for choosing the non-rewarded corridor (= false).

**Trial Number**	**Date**	**Test Series**	**correct/false**
1	06.05.2016	TS1a	f
2	06.05.2016	TS1a	c
3	06.05.2016	TS1a	f
4	06.05.2016	TS1a	c
5	06.05.2016	TS1a	f
6	06.05.2016	TS1a	c
7	06.05.2016	TS1a	c
8	06.05.2016	TS1a	c
9	06.05.2016	TS1a	f
10	06.05.2016	TS1a	f
11	08.05.2016	TS1a	f
12	08.05.2016	TS1a	f
13	08.05.2016	TS1a	c
14	08.05.2016	TS1a	f
15	08.05.2016	TS1a	c
16	08.05.2016	TS1a	f
17	08.05.2016	TS1a	c
18	08.05.2016	TS1a	f
19	08.05.2016	TS1a	c
20	08.05.2016	TS1a	f
21	10.05.2016	TS1a	f
22	10.05.2016	TS1a	f
23	10.05.2016	TS1a	f
24	10.05.2016	TS1a	c
25	10.05.2016	TS1a	c
26	10.05.2016	TS1a	c
27	10.05.2016	TS1a	f
28	10.05.2016	TS1a	c
29	10.05.2016	TS1a	c
30	10.05.2016	TS1a	c
31	11.05.2016	TS1a	f
32	11.05.2016	TS1a	c
33	11.05.2016	TS1a	f
34	11.05.2016	TS1a	f
35	11.05.2016	TS1a	f
36	11.05.2016	TS1a	c
37	11.05.2016	TS1a	c
38	11.05.2016	TS1a	c
39	11.05.2016	TS1a	f
40	11.05.2016	TS1a	c
41	11.05.2016	TS1a	f

42	11.05.2016	TS1a	c
43	11.05.2016	TS1a	f
44	11.05.2016	TS1a	f
45	11.05.2016	TS1a	c
46	12.05.2016	TS1a	f
47	12.05.2016	TS1a	c
48	12.05.2016	TS1a	f
49	12.05.2016	TS1a	c
50	12.05.2016	TS1a	c
51	12.05.2016	TS1a	f
52	12.05.2016	TS1a	c
53	12.05.2016	TS1a	c
54	12.05.2016	TS1a	c
55	12.05.2016	TS1a	c
56	12.05.2016	TS1a	c
57	12.05.2016	TS1a	c
58	12.05.2016	TS1a	c
59	12.05.2016	TS1a	c
60	12.05.2016	TS1a	c
61	13.05.2016	TS1a	c
62	13.05.2016	TS1a	c
63	13.05.2016	TS1a	c
64	13.05.2016	TS1a	c
65	13.05.2016	TS1a	f
66	13.05.2016	TS1a	c
67	13.05.2016	TS1a	c
68	13.05.2016	TS1a	f
69	13.05.2016	TS1a	c
70	13.05.2016	TS1a	c
71	13.05.2016	TS1a	c
72	13.05.2016	TS1a	c
73	13.05.2016	TS1a	c
74	13.05.2016	TS1a	c
75	18.05.2016	TS1a	f
76	18.05.2016	TS1a	c
77	18.05.2016	TS1a	c
78	18.05.2016	TS1a	f
79	18.05.2016	TS1a	f
80	18.05.2016	TS1a	f
81	19.05.2016	TS1a	f
82	19.05.2016	TS1a	c
83	19.05.2016	TS1a	c
84	19.05.2016	TS1a	c
85	19.05.2016	TS1a	c
86	19.05.2016	TS1a	c

87	19.05.2016	TS1a	c
88	19.05.2016	TS1a	c
89	19.05.2016	TS1a	c
90	19.05.2016	TS1a	c
91	19.05.2016	TS1a	c
92	19.05.2016	TS1a	c
93	19.05.2016	TS1a	c
94	19.05.2016	TS1a	c
95	19.05.2016	TS1a	c
96	20.05.2016	TS1a	f
97	20.05.2016	TS1a	f
98	20.05.2016	TS1a	c
99	20.05.2016	TS1a	c
100	20.05.2016	TS1a	c
101	20.05.2016	TS1a	c
102	20.05.2016	TS1a	c
103	20.05.2016	TS1a	c
104	20.05.2016	TS1a	c
105	20.05.2016	TS1a	c
106	20.05.2016	TS1a	c
107	20.05.2016	TS1a	c
108	20.05.2016	TS1a	c
109	20.05.2016	TS1a	c
110	20.05.2016	TS1a	c
111	23.05.2016	TS1a	f
112	23.05.2016	TS1a	c
113	23.05.2016	TS1a	c
114	23.05.2016	TS1a	c
115	23.05.2016	TS1a	c
116	23.05.2016	TS1a	c
117	23.05.2016	TS1a	c
118	23.05.2016	TS1a	c
119	23.05.2016	TS1a	c
120	23.05.2016	TS1a	c
121	23.05.2016	TS1a	f
122	23.05.2016	TS1a	f
123	23.05.2016	TS1a	c
124	23.05.2016	TS1a	c
125	23.05.2016	TS1a	c
126	24.05.2016	TS1a	f
127	24.05.2016	TS1a	c
128	24.05.2016	TS1a	c
129	24.05.2016	TS1a	c
130	24.05.2016	TS1a	c
131	24.05.2016	TS1a	f

132	24.05.2016	TS1a	c
133	24.05.2016	TS1a	c
134	24.05.2016	TS1a	c
135	24.05.2016	TS1a	c
136	24.05.2016	TS1a	c
137	24.05.2016	TS1a	c
138	24.05.2016	TS1a	c
139	24.05.2016	TS1a	c
140	24.05.2016	TS1a	c
141	25.05.2016	TS1b	f
142	25.05.2016	TS1b	f
143	25.05.2016	TS1b	f
144	25.05.2016	TS1b	f
145	25.05.2016	TS1b	c
146	25.05.2016	TS1b	c
147	25.05.2016	TS1b	c
148	25.05.2016	TS1b	c
149	25.05.2016	TS1b	c
150	25.05.2016	TS1b	c
151	30.05.2016	TS1b	c
152	30.05.2016	TS1b	f
153	30.05.2016	TS1b	c
154	30.05.2016	TS1b	c
155	30.05.2016	TS1b	c
156	30.05.2016	TS1b	c
157	30.05.2016	TS1b	c
158	30.05.2016	TS1b	f
159	30.05.2016	TS1b	c
160	30.05.2016	TS1b	c
161	02.06.2016	TS1b	f
162	02.06.2016	TS1b	c
163	02.06.2016	TS1b	f
164	02.06.2016	TS1b	c
165	02.06.2016	TS1b	f
166	02.06.2016	TS1b	c
167	02.06.2016	TS1b	c
168	02.06.2016	TS1b	c
169	02.06.2016	TS1b	f
170	02.06.2016	TS1b	c
171	02.06.2016	TS1b	c
172	02.06.2016	TS1b	c
173	02.06.2016	TS1b	c
174	02.06.2016	TS1b	c
175	02.06.2016	TS1b	c
176	03.06.2016	TS1b	f

177	03.06.2016	TS1b	c
178	03.06.2016	TS1b	c
179	03.06.2016	TS1b	c
180	03.06.2016	TS1b	c
181	03.06.2016	TS1b	c
182	03.06.2016	TS1b	c
183	03.06.2016	TS1b	c
184	03.06.2016	TS1b	f
185	03.06.2016	TS1b	c
186	03.06.2016	TS1b	c
187	03.06.2016	TS1b	c
188	03.06.2016	TS1b	c
189	03.06.2016	TS1b	c
190	03.06.2016	TS1b	c
191	06.06.2016	TS1c	c
192	06.06.2016	TS1c	c
193	06.06.2016	TS1c	c
194	06.06.2016	TS1c	c
195	06.06.2016	TS1c	c
196	06.06.2016	TS1c	c
197	06.06.2016	TS1c	f
198	06.06.2016	TS1c	c
199	06.06.2016	TS1c	c
200	06.06.2016	TS1c	c
201	06.06.2016	TS1c	c
202	06.06.2016	TS1c	c
203	06.06.2016	TS1c	f
204	06.06.2016	TS1c	c
205	06.06.2016	TS1c	c
206	07.06.2016	TS1c	c
207	07.06.2016	TS1c	c
208	07.06.2016	TS1c	f
209	07.06.2016	TS1c	c
210	07.06.2016	TS1c	c
211	07.06.2016	TS1c	c
212	07.06.2016	TS1c	c
213	07.06.2016	TS1c	c
214	07.06.2016	TS1c	c
215	07.06.2016	TS1c	c
216	07.06.2016	TS1c	c
217	07.06.2016	TS1c	c
218	07.06.2016	TS1c	c
219	07.06.2016	TS1c	c
220	07.06.2016	TS1c	c
221	08.06.2016	TS1c	f

222	08.06.2016	TS1c	f
223	08.06.2016	TS1c	c
224	08.06.2016	TS1c	c
225	08.06.2016	TS1c	c
226	08.06.2016	TS1c	c
227	08.06.2016	TS1c	c
228	08.06.2016	TS1c	f
229	08.06.2016	TS1c	c
230	08.06.2016	TS1c	f
231	08.06.2016	TS1c	c
232	08.06.2016	TS1c	c
233	08.06.2016	TS1c	c
234	08.06.2016	TS1c	f
235	08.06.2016	TS1c	f
236	09.06.2016	TS1c	f
237	09.06.2016	TS1c	f
238	09.06.2016	TS1c	c
239	09.06.2016	TS1c	f
240	09.06.2016	TS1c	c
241	09.06.2016	TS1c	c
242	09.06.2016	TS1c	c
243	09.06.2016	TS1c	c
244	09.06.2016	TS1c	c
245	09.06.2016	TS1c	c
246	09.06.2016	TS1c	f
247	09.06.2016	TS1c	c
248	09.06.2016	TS1c	c
249	09.06.2016	TS1c	c
250	09.06.2016	TS1c	c
251	10.06.2016	TS1c	c
252	10.06.2016	TS1c	f
253	10.06.2016	TS1c	c
254	10.06.2016	TS1c	c
255	10.06.2016	TS1c	c
256	10.06.2016	TS1c	f
257	10.06.2016	TS1c	f
258	10.06.2016	TS1c	c
259	10.06.2016	TS1c	c
260	10.06.2016	TS1c	c
261	10.06.2016	TS1c	c
262	10.06.2016	TS1c	c
263	10.06.2016	TS1c	c
264	10.06.2016	TS1c	c
265	10.06.2016	TS1c	c
266	13.06.2016	TS2	c

267	13.06.2016	TS2	c
268	13.06.2016	TS2	c
269	13.06.2016	TS2	c
270	13.06.2016	TS2	c
271	13.06.2016	TS2	c
272	13.06.2016	TS2	c
273	13.06.2016	TS2	c
274	13.06.2016	TS2	c
275	13.06.2016	TS2	c
276	13.06.2016	TS2	c
277	13.06.2016	TS2	c
278	13.06.2016	TS2	c
279	13.06.2016	TS2	c
280	13.06.2016	TS2	c
281	14.06.2016	TS2	c
282	14.06.2016	TS2	c
283	14.06.2016	TS2	c
284	14.06.2016	TS2	c
285	14.06.2016	TS2	c
286	14.06.2016	TS2	c
287	14.06.2016	TS2	f
288	14.06.2016	TS2	f
289	14.06.2016	TS2	f
290	14.06.2016	TS2	c
291	14.06.2016	TS2	c
292	14.06.2016	TS2	c
293	14.06.2016	TS2	c
294	14.06.2016	TS2	c
295	14.06.2016	TS2	c
296	15.06.2016	TS3	f
297	15.06.2016	TS3	c
298	15.06.2016	TS3	f
299	15.06.2016	TS3	c
300	15.06.2016	TS3	c
301	15.06.2016	TS3	c
302	15.06.2016	TS3	c
303	15.06.2016	TS3	c
304	15.06.2016	TS3	f
305	15.06.2016	TS3	c
306	15.06.2016	TS3	c
307	15.06.2016	TS3	c
308	15.06.2016	TS3	c
309	15.06.2016	TS3	c
310	15.06.2016	TS3	c
311	16.06.2016	TS3	c

312	16.06.2016	TS3	c
313	16.06.2016	TS3	f
314	16.06.2016	TS3	f
315	16.06.2016	TS3	c
316	16.06.2016	TS3	c
317	16.06.2016	TS3	c
318	16.06.2016	TS3	c
319	16.06.2016	TS3	c
320	16.06.2016	TS3	c
321	16.06.2016	TS3	c
322	16.06.2016	TS3	c
323	16.06.2016	TS3	c
324	16.06.2016	TS3	c
325	16.06.2016	TS3	c
326	17.06.2016	TS3	f
327	17.06.2016	TS3	f
328	17.06.2016	TS3	c
329	17.06.2016	TS3	c
330	17.06.2016	TS3	c
331	17.06.2016	TS3	c
332	17.06.2016	TS3	f
333	17.06.2016	TS3	f
334	17.06.2016	TS3	c
335	17.06.2016	TS3	c
336	17.06.2016	TS3	c
337	17.06.2016	TS3	c
338	17.06.2016	TS3	c
339	17.06.2016	TS3	c
340	17.06.2016	TS3	c
341	20.06.2016	TS3	f
342	20.06.2016	TS3	c
343	20.06.2016	TS3	f
344	20.06.2016	TS3	c
345	20.06.2016	TS3	c
346	20.06.2016	TS3	c
347	20.06.2016	TS3	c
348	20.06.2016	TS3	c
349	20.06.2016	TS3	c
350	20.06.2016	TS3	c
351	20.06.2016	TS3	c
352	20.06.2016	TS3	c
353	20.06.2016	TS3	c
354	20.06.2016	TS3	c
355	20.06.2016	TS3	c

Table S4: Raw data of GND in October-November 2016. A “c” stands for choosing the rewarded corridor (= correct). An “f” stands for choosing the non-rewarded corridor (= false).

Trial Number	Date	Test Series	correct/false
1	04.10.2016	TS1a	c
2	04.10.2016	TS1a	c
3	04.10.2016	TS1a	f
4	04.10.2016	TS1a	c
5	04.10.2016	TS1a	c
6	04.10.2016	TS1a	c
7	04.10.2016	TS1a	c
8	04.10.2016	TS1a	c
9	04.10.2016	TS1a	c
10	04.10.2016	TS1a	c
11	05.10.2016	TS1a	c
12	05.10.2016	TS1a	c
13	05.10.2016	TS1a	c
14	05.10.2016	TS1a	c
15	05.10.2016	TS1a	c
16	05.10.2016	TS1a	c
17	05.10.2016	TS1a	c
18	05.10.2016	TS1a	c
19	05.10.2016	TS1a	c
20	06.10.2016	TS1a	c
21	06.10.2016	TS1a	c
22	06.10.2016	TS1a	c
23	06.10.2016	TS1a	c
24	06.10.2016	TS1a	c
25	06.10.2016	TS1a	c
26	06.10.2016	TS1a	c
27	06.10.2016	TS1a	c
28	06.10.2016	TS1a	c
29	06.10.2016	TS1a	c
30	07.10.2016	TS1a	f
31	07.10.2016	TS1a	c
32	07.10.2016	TS1a	c
33	07.10.2016	TS1a	c
34	07.10.2016	TS1a	c
35	07.10.2016	TS1a	c
36	07.10.2016	TS1a	c
37	10.10.2016	TS1a	f
38	10.10.2016	TS1a	c
39	10.10.2016	TS1a	c
40	10.10.2016	TS1a	c
41	10.10.2016	TS1a	c

42	10.10.2016	TS1a	c
43	10.10.2016	TS1a	c
44	10.10.2016	TS1a	c
45	10.10.2016	TS1a	c
46	10.10.2016	TS1a	c
47	10.10.2016	TS1a	c
48	10.10.2016	TS1a	c
49	10.10.2016	TS1a	c
50	10.10.2016	TS1a	c
51	10.10.2016	TS1a	c
52	11.10.2016	TS1a	c
53	11.10.2016	TS1a	c
54	11.10.2016	TS1a	c
55	11.10.2016	TS1a	c
56	11.10.2016	TS1a	c
57	11.10.2016	TS1a	c
58	11.10.2016	TS1a	c
59	11.10.2016	TS1a	c
60	11.10.2016	TS1a	c
61	11.10.2016	TS1a	c
62	11.10.2016	TS1a	c
63	11.10.2016	TS1a	c
64	11.10.2016	TS1a	c
65	11.10.2016	TS1a	c
66	11.10.2016	TS1a	c
67	12.10.2016	TS1a	f
68	12.10.2016	TS1a	c
69	12.10.2016	TS1a	f
70	12.10.2016	TS1a	c
71	12.10.2016	TS1a	c
72	12.10.2016	TS1a	c
73	12.10.2016	TS1a	c
74	12.10.2016	TS1a	c
75	12.10.2016	TS1a	c
76	12.10.2016	TS1a	c
77	12.10.2016	TS1a	c
78	12.10.2016	TS1a	c
79	12.10.2016	TS1a	c
80	12.10.2016	TS1a	c
81	12.10.2016	TS1a	c
82	13.10.2016	TS1a	f
83	13.10.2016	TS1a	c
84	13.10.2016	TS1a	c
85	14.10.2016	TS1a	c
86	17.10.2016	TS1a	c

87	17.10.2016	TS1a	c
88	17.10.2016	TS1a	f
89	17.10.2016	TS1a	c
90	17.10.2016	TS1a	c
91	17.10.2016	TS1a	c
92	17.10.2016	TS1a	c
93	17.10.2016	TS1a	c
94	17.10.2016	TS1a	c
95	17.10.2016	TS1a	c
96	17.10.2016	TS1a	c
97	17.10.2016	TS1a	c
98	17.10.2016	TS1a	c
99	17.10.2016	TS1a	c
100	17.10.2016	TS1a	c
101	18.10.2016	TS1b	c
102	18.10.2016	TS1b	c
103	18.10.2016	TS1b	f
104	18.10.2016	TS1b	c
105	18.10.2016	TS1b	c
106	18.10.2016	TS1b	c
107	18.10.2016	TS1b	c
108	18.10.2016	TS1b	c
109	18.10.2016	TS1b	c
110	18.10.2016	TS1b	c
111	18.10.2016	TS1b	c
112	18.10.2016	TS1b	c
113	18.10.2016	TS1b	c
114	18.10.2016	TS1b	c
115	18.10.2016	TS1b	c
116	20.10.2016	TS1b	c
117	20.10.2016	TS1b	c
118	20.10.2016	TS1b	f
119	20.10.2016	TS1b	c
120	20.10.2016	TS1b	c
121	20.10.2016	TS1b	c
122	20.10.2016	TS1b	c
123	20.10.2016	TS1b	c
124	20.10.2016	TS1b	c
125	20.10.2016	TS1b	c
126	20.10.2016	TS1b	c
127	20.10.2016	TS1b	c
128	20.10.2016	TS1b	c
129	20.10.2016	TS1b	c
130	20.10.2016	TS1b	c
131	21.10.2016	TS1c	c

132	21.10.2016	TS1c	c
133	21.10.2016	TS1c	c
134	21.10.2016	TS1c	c
135	21.10.2016	TS1c	c
136	21.10.2016	TS1c	c
137	21.10.2016	TS1c	c
138	21.10.2016	TS1c	c
139	21.10.2016	TS1c	c
140	21.10.2016	TS1c	c
141	21.10.2016	TS1c	c
142	21.10.2016	TS1c	c
143	21.10.2016	TS1c	c
144	21.10.2016	TS1c	c
145	21.10.2016	TS1c	c
146	28.10.2016	TS1c	c
147	28.10.2016	TS1c	c
148	28.10.2016	TS1c	c
149	28.10.2016	TS1c	c
150	28.10.2016	TS1c	c
151	28.10.2016	TS1c	c
152	28.10.2016	TS1c	f
153	28.10.2016	TS1c	c
154	28.10.2016	TS1c	c
155	28.10.2016	TS1c	c
156	28.10.2016	TS1c	c
157	28.10.2016	TS1c	c
158	28.10.2016	TS1c	c
159	28.10.2016	TS1c	c
160	28.10.2016	TS1c	c
161	31.10.2016	TS1c	f
162	31.10.2016	TS1c	c
163	31.10.2016	TS1c	c
164	31.10.2016	TS1c	f
165	31.10.2016	TS1c	c
166	31.10.2016	TS1c	c
167	31.10.2016	TS1c	c
168	31.10.2016	TS1c	f
169	31.10.2016	TS1c	c
170	31.10.2016	TS1c	c
171	31.10.2016	TS1c	c
172	31.10.2016	TS1c	c
173	31.10.2016	TS1c	c
174	31.10.2016	TS1c	c
175	31.10.2016	TS1c	c
176	01.11.2016	TS1c	f

177	01.11.2016	TS1c	c
178	01.11.2016	TS1c	f
179	01.11.2016	TS1c	c
180	01.11.2016	TS1c	c
181	01.11.2016	TS1c	c
182	01.11.2016	TS1c	c
183	01.11.2016	TS1c	c
184	01.11.2016	TS1c	c
185	01.11.2016	TS1c	c
186	01.11.2016	TS1c	f
187	01.11.2016	TS1c	c
188	01.11.2016	TS1c	c
189	01.11.2016	TS1c	c
190	01.11.2016	TS1c	c
191	02.11.2016	TS2	c
192	02.11.2016	TS2	c
193	02.11.2016	TS2	f
194	02.11.2016	TS2	c
195	02.11.2016	TS2	c
196	02.11.2016	TS2	c
197	02.11.2016	TS2	c
198	02.11.2016	TS2	c
199	02.11.2016	TS2	c
200	02.11.2016	TS2	c
201	02.11.2016	TS2	c
202	02.11.2016	TS2	c
203	02.11.2016	TS2	c
204	02.11.2016	TS2	c
205	02.11.2016	TS2	c
206	03.11.2016	TS2	c
207	03.11.2016	TS2	c
208	03.11.2016	TS2	f
209	03.11.2016	TS2	c
210	03.11.2016	TS2	c
211	03.11.2016	TS2	c
212	03.11.2016	TS2	c
213	03.11.2016	TS2	c
214	03.11.2016	TS2	c
215	03.11.2016	TS2	c
216	03.11.2016	TS2	c
217	03.11.2016	TS2	c
218	03.11.2016	TS2	c
219	03.11.2016	TS2	c
220	03.11.2016	TS2	c
221	04.11.2016	TS3	c

222	04.11.2016	TS3	c
223	04.11.2016	TS3	c
224	04.11.2016	TS3	c
225	04.11.2016	TS3	f
226	04.11.2016	TS3	c
227	04.11.2016	TS3	c
228	04.11.2016	TS3	c
229	04.11.2016	TS3	c
230	04.11.2016	TS3	c
231	04.11.2016	TS3	f
232	04.11.2016	TS3	c
233	04.11.2016	TS3	c
234	04.11.2016	TS3	c
235	04.11.2016	TS3	c
236	07.11.2016	TS3	c
237	07.11.2016	TS3	c
238	07.11.2016	TS3	c
239	07.11.2016	TS3	c
240	07.11.2016	TS3	c
241	07.11.2016	TS3	c
242	07.11.2016	TS3	c
243	07.11.2016	TS3	c
244	07.11.2016	TS3	f
245	07.11.2016	TS3	c
246	07.11.2016	TS3	c
247	07.11.2016	TS3	c
248	07.11.2016	TS3	c
249	07.11.2016	TS3	c
250	07.11.2016	TS3	c

Table S5: Raw data of GIN in October-November 2016. A “c” stands for choosing the rewarded corridor (= correct). An “f” stands for choosing the non-rewarded corridor (= false).

**Trial Number**	**Date**	**Test Series**	**correct/false**
1	04.10.2016	TS1a	c
2	04.10.2016	TS1a	f
3	04.10.2016	TS1a	c
4	04.10.2016	TS1a	c
5	04.10.2016	TS1a	c
6	04.10.2016	TS1a	c
7	04.10.2016	TS1a	f
8	04.10.2016	TS1a	c

9	04.10.2016	TS1a	c
10	04.10.2016	TS1a	c
11	05.10.2016	TS1a	c
12	05.10.2016	TS1a	c
13	05.10.2016	TS1a	f
14	05.10.2016	TS1a	f
15	05.10.2016	TS1a	c
16	05.10.2016	TS1a	c
17	05.10.2016	TS1a	f
18	05.10.2016	TS1a	c
19	05.10.2016	TS1a	c
20	06.10.2016	TS1a	c
21	06.10.2016	TS1a	c
22	06.10.2016	TS1a	c
23	06.10.2016	TS1a	c
24	06.10.2016	TS1a	f
25	06.10.2016	TS1a	c
26	06.10.2016	TS1a	c
27	10.10.2016	TS1a	c
28	10.10.2016	TS1a	c
29	10.10.2016	TS1a	c
30	11.10.2016	TS1a	c
31	12.10.2016	TS1a	c
32	12.10.2016	TS1a	c
33	12.10.2016	TS1a	c
34	12.10.2016	TS1a	c
35	12.10.2016	TS1a	f
36	12.10.2016	TS1a	c
37	12.10.2016	TS1a	c
38	12.10.2016	TS1a	c
39	12.10.2016	TS1a	c
40	12.10.2016	TS1a	c
41	13.10.2016	TS1a	c
42	13.10.2016	TS1a	c
43	13.10.2016	TS1a	c
44	13.10.2016	TS1a	c
45	14.10.2016	TS1a	f
46	14.10.2016	TS1a	f
47	14.10.2016	TS1a	c
48	14.10.2016	TS1a	c
49	14.10.2016	TS1a	c
50	17.10.2016	TS1a	c
51	17.10.2016	TS1a	c
52	17.10.2016	TS1a	c
53	17.10.2016	TS1a	c

54	17.10.2016	TS1a	c
55	17.10.2016	TS1a	c
56	17.10.2016	TS1a	c
57	17.10.2016	TS1a	c
58	17.10.2016	TS1a	f
59	17.10.2016	TS1a	c
60	18.10.2016	TS1a	c
61	18.10.2016	TS1a	c
62	18.10.2016	TS1a	c
63	18.10.2016	TS1a	c
64	18.10.2016	TS1a	f
65	18.10.2016	TS1a	c
66	18.10.2016	TS1a	c
67	18.10.2016	TS1a	c
68	20.10.2016	TS1a	c
69	20.10.2016	TS1a	c
70	20.10.2016	TS1a	c
71	20.10.2016	TS1a	c
72	20.10.2016	TS1a	c
73	20.10.2016	TS1a	c
74	20.10.2016	TS1a	c
75	20.10.2016	TS1a	c
76	20.10.2016	TS1a	f
77	20.10.2016	TS1a	c
78	20.10.2016	TS1a	c
79	20.10.2016	TS1a	c
80	20.10.2016	TS1a	c
81	20.10.2016	TS1a	c
82	20.10.2016	TS1a	c
83	21.10.2016	TS1a	c
84	21.10.2016	TS1a	c
85	21.10.2016	TS1a	c
86	21.10.2016	TS1a	c
87	21.10.2016	TS1a	c
88	21.10.2016	TS1a	c
89	21.10.2016	TS1a	c
90	21.10.2016	TS1a	c
91	21.10.2016	TS1a	c
92	21.10.2016	TS1a	c
93	21.10.2016	TS1a	c
94	21.10.2016	TS1a	c
95	24.10.2016	TS1b	c
96	24.10.2016	TS1b	c
97	26.10.2016	TS1b	f
98	26.10.2016	TS1b	c

99	26.10.2016	TS1b	c
100	26.10.2016	TS1b	c
101	26.10.2016	TS1b	c
102	26.10.2016	TS1b	c
103	27.10.2016	TS1b	c
104	27.10.2016	TS1b	c
105	27.10.2016	TS1b	f
106	27.10.2016	TS1b	c
107	27.10.2016	TS1b	c
108	27.10.2016	TS1b	c
109	27.10.2016	TS1b	c
110	27.10.2016	TS1b	c
111	27.10.2016	TS1b	c
112	27.10.2016	TS1b	c
113	28.10.2016	TS1b	f
114	28.10.2016	TS1b	f
115	31.10.2016	TS1b	c
116	31.10.2016	TS1b	c
117	31.10.2016	TS1b	c
118	31.10.2016	TS1b	c
119	31.10.2016	TS1b	c
120	01.11.2016	TS1b	c
121	01.11.2016	TS1b	c
122	01.11.2016	TS1b	c
123	01.11.2016	TS1b	c
124	01.11.2016	TS1b	f
125	01.11.2016	TS1b	f
126	01.11.2016	TS1b	c
127	01.11.2016	TS1b	c
128	01.11.2016	TS1b	c
129	02.11.2016	TS1b	c
130	02.11.2016	TS1b	c
131	02.11.2016	TS1b	c
132	02.11.2016	TS1b	c
133	02.11.2016	TS1b	c
134	02.11.2016	TS1b	c
135	02.11.2016	TS1b	c
136	02.11.2016	TS1b	c
137	03.11.2016	TS1c	f
138	03.11.2016	TS1c	c
139	03.11.2016	TS1c	f
140	03.11.2016	TS1c	c
141	03.11.2016	TS1c	c
142	03.11.2016	TS1c	f
143	03.11.2016	TS1c	c

144	03.11.2016	TS1c	c
145	03.11.2016	TS1c	f
146	04.11.2016	TS1c	f
147	04.11.2016	TS1c	c
148	04.11.2016	TS1c	c
149	04.11.2016	TS1c	c
150	04.11.2016	TS1c	c
151	04.11.2016	TS1c	c
152	04.11.2016	TS1c	c
153	07.11.2016	TS1c	c
154	07.11.2016	TS1c	c
155	07.11.2016	TS1c	c
156	07.11.2016	TS1c	c
157	07.11.2016	TS1c	c
158	07.11.2016	TS1c	c
159	08.11.2016	TS1c	c
160	08.11.2016	TS1c	c
161	08.11.2016	TS1c	c
162	08.11.2016	TS1c	c
163	08.11.2016	TS1c	c
164	08.11.2016	TS1c	c
165	09.11.2016	TS1c	f
166	09.11.2016	TS1c	f
167	09.11.2016	TS1c	c
168	09.11.2016	TS1c	c
169	09.11.2016	TS1c	f
170	09.11.2016	TS1c	c
171	09.11.2016	TS1c	c
172	09.11.2016	TS1c	c
173	09.11.2016	TS1c	c
174	09.11.2016	TS1c	c
175	09.11.2016	TS1c	c
176	09.11.2016	TS1c	c
177	11.11.2016	TS2	c
178	11.11.2016	TS2	c
179	11.11.2016	TS2	c
180	11.11.2016	TS2	c
181	11.11.2016	TS2	c
182	11.11.2016	TS2	c
183	14.11.2016	TS2	f
184	14.11.2016	TS2	c
185	14.11.2016	TS2	c
186	14.11.2016	TS2	f
187	14.11.2016	TS2	c
188	14.11.2016	TS2	c

189	14.11.2016	TS2	c
190	14.11.2016	TS2	c
191	14.11.2016	TS2	c
192	14.11.2016	TS2	c
193	14.11.2016	TS2	c
194	14.11.2016	TS2	c
195	14.11.2016	TS2	c
196	14.11.2016	TS2	c
197	15.11.2016	TS3	c
198	15.11.2016	TS3	c
199	15.11.2016	TS3	c
200	15.11.2016	TS3	c
201	15.11.2016	TS3	c
202	15.11.2016	TS3	c
203	15.11.2016	TS3	c
204	15.11.2016	TS3	c
205	15.11.2016	TS3	c
206	15.11.2016	TS3	c
207	15.11.2016	TS3	c
208	15.11.2016	TS3	c
209	16.11.2016	TS3	f
210	16.11.2016	TS3	f
211	16.11.2016	TS3	c
212	16.11.2016	TS3	f
213	16.11.2016	TS3	f
214	16.11.2016	TS3	c
215	16.11.2016	TS3	c
216	16.11.2016	TS3	c
217	16.11.2016	TS3	c
218	16.11.2016	TS3	c
219	21.11.2016	TS3	c
220	21.11.2016	TS3	c
221	21.11.2016	TS3	c
222	21.11.2016	TS3	f
223	21.11.2016	TS3	c
224	21.11.2016	TS3	c
225	21.11.2016	TS3	f
226	21.11.2016	TS3	c
227	21.11.2016	TS3	c
228	21.11.2016	TS3	c
229	21.11.2016	TS3	f
230	22.11.2016	TS3	c
231	22.11.2016	TS3	c
232	22.11.2016	TS3	c
233	22.11.2016	TS3	c

234	22.11.2016	TS3	c
235	22.11.2016	TS3	c
236	22.11.2016	TS3	c
237	22.11.2016	TS3	c
238	22.11.2016	TS3	f
239	22.11.2016	TS3	c
240	22.11.2016	TS3	c
241	22.11.2016	TS3	c
242	22.11.2016	TS3	c
243	22.11.2016	TS3	c

Table S6: Raw data of PUM in October-November 2016. A “c” stands for choosing the rewarded corridor (= correct). An “f” stands for choosing the non-rewarded corridor (= false).

**Trial Number**	**Date**	**Test Series**	**correct/false**
1	04.10.2016	TS1a	c
2	04.10.2016	TS1a	f
3	04.10.2016	TS1a	c
4	04.10.2016	TS1a	f
5	04.10.2016	TS1a	f
6	04.10.2016	TS1a	c
7	04.10.2016	TS1a	f
8	04.10.2016	TS1a	f
9	04.10.2016	TS1a	c
10	04.10.2016	TS1a	c
11	05.10.2016	TS1a	f
12	05.10.2016	TS1a	c
13	05.10.2016	TS1a	f
14	05.10.2016	TS1a	f
15	05.10.2016	TS1a	c
16	05.10.2016	TS1a	c
17	05.10.2016	TS1a	f
18	05.10.2016	TS1a	c
19	05.10.2016	TS1a	c
20	05.10.2016	TS1a	c
21	06.10.2016	TS1a	c
22	06.10.2016	TS1a	f
23	06.10.2016	TS1a	f
24	06.10.2016	TS1a	c
25	06.10.2016	TS1a	f
26	06.10.2016	TS1a	c
27	06.10.2016	TS1a	c

28	06.10.2016	TS1a	c
29	06.10.2016	TS1a	f
30	06.10.2016	TS1a	c
31	06.10.2016	TS1a	c
32	06.10.2016	TS1a	c
33	06.10.2016	TS1a	c
34	06.10.2016	TS1a	f
35	06.10.2016	TS1a	f
36	07.10.2016	TS1a	c
37	07.10.2016	TS1a	f
38	07.10.2016	TS1a	f
39	07.10.2016	TS1a	f
40	07.10.2016	TS1a	f
41	07.10.2016	TS1a	c
42	07.10.2016	TS1a	f
43	07.10.2016	TS1a	c
44	07.10.2016	TS1a	f
45	07.10.2016	TS1a	f
46	07.10.2016	TS1a	f
47	07.10.2016	TS1a	c
48	07.10.2016	TS1a	c
49	07.10.2016	TS1a	f
50	07.10.2016	TS1a	c
51	10.10.2016	TS1a	c
52	10.10.2016	TS1a	f
53	10.10.2016	TS1a	c
54	10.10.2016	TS1a	c
55	10.10.2016	TS1a	f
56	10.10.2016	TS1a	c
57	10.10.2016	TS1a	f
58	10.10.2016	TS1a	c
59	10.10.2016	TS1a	c
60	11.10.2016	TS1a	c
61	11.10.2016	TS1a	c
62	11.10.2016	TS1a	c
63	11.10.2016	TS1a	c
64	11.10.2016	TS1a	f
65	11.10.2016	TS1a	c
66	11.10.2016	TS1a	c
67	11.10.2016	TS1a	c
68	11.10.2016	TS1a	f
69	11.10.2016	TS1a	c
70	11.10.2016	TS1a	c
71	11.10.2016	TS1a	c
72	11.10.2016	TS1a	f

73	11.10.2016	TS1a	c
74	12.10.2016	TS1a	f
75	12.10.2016	TS1a	f
76	12.10.2016	TS1a	f
77	12.10.2016	TS1a	c
78	12.10.2016	TS1a	c
79	12.10.2016	TS1a	c
80	12.10.2016	TS1a	f
81	12.10.2016	TS1a	c
82	12.10.2016	TS1a	c
83	12.10.2016	TS1a	c
84	12.10.2016	TS1a	c
85	12.10.2016	TS1a	f
86	12.10.2016	TS1a	f
87	12.10.2016	TS1a	c
88	12.10.2016	TS1a	c
89	13.10.2016	TS1a	c
90	13.10.2016	TS1a	f
91	13.10.2016	TS1a	f
92	13.10.2016	TS1a	c
93	13.10.2016	TS1a	c
94	13.10.2016	TS1a	c
95	13.10.2016	TS1a	c
96	13.10.2016	TS1a	c
97	13.10.2016	TS1a	f
98	13.10.2016	TS1a	c
99	13.10.2016	TS1a	c
100	13.10.2016	TS1a	f
101	13.10.2016	TS1a	c
102	13.10.2016	TS1a	c
103	13.10.2016	TS1a	c
104	14.10.2016	TS1a	c
105	14.10.2016	TS1a	c
106	14.10.2016	TS1a	c
107	14.10.2016	TS1a	c
108	14.10.2016	TS1a	f
109	14.10.2016	TS1a	c
110	14.10.2016	TS1a	c
111	14.10.2016	TS1a	c
112	14.10.2016	TS1a	c
113	14.10.2016	TS1a	c
114	14.10.2016	TS1a	c
115	14.10.2016	TS1a	c
116	14.10.2016	TS1a	f
117	14.10.2016	TS1a	c

118	14.10.2016	TS1a	c
119	17.10.2016	TS1a	c
120	17.10.2016	TS1a	c
121	17.10.2016	TS1a	c
122	17.10.2016	TS1a	c
123	17.10.2016	TS1a	f
124	17.10.2016	TS1a	c
125	17.10.2016	TS1a	f
126	17.10.2016	TS1a	f
127	17.10.2016	TS1a	c
128	17.10.2016	TS1a	c
129	17.10.2016	TS1a	c
130	17.10.2016	TS1a	c
131	17.10.2016	TS1a	f
132	17.10.2016	TS1a	f
133	17.10.2016	TS1a	c
134	18.10.2016	TS1a	c
135	18.10.2016	TS1a	f
136	18.10.2016	TS1a	c
137	18.10.2016	TS1a	c
138	18.10.2016	TS1a	c
139	18.10.2016	TS1a	c
140	18.10.2016	TS1a	c
141	18.10.2016	TS1a	f
142	18.10.2016	TS1a	c
143	18.10.2016	TS1a	c
144	19.10.2016	TS1a	c
145	19.10.2016	TS1a	c
146	19.10.2016	TS1a	c
147	19.10.2016	TS1a	c
148	19.10.2016	TS1a	c
149	19.10.2016	TS1a	c
150	19.10.2016	TS1a	c
151	19.10.2016	TS1a	c
152	19.10.2016	TS1a	f
153	19.10.2016	TS1a	c
154	19.10.2016	TS1a	c
155	19.10.2016	TS1a	f
156	19.10.2016	TS1a	c
157	19.10.2016	TS1a	c
158	19.10.2016	TS1a	f
159	21.10.2016	TS1b	c
160	21.10.2016	TS1b	f
161	21.10.2016	TS1b	f
162	21.10.2016	TS1b	c

163	21.10.2016	TS1b	c
164	21.10.2016	TS1b	c
165	21.10.2016	TS1b	f
166	21.10.2016	TS1b	f
167	21.10.2016	TS1b	f
168	21.10.2016	TS1b	c
169	21.10.2016	TS1b	c
170	21.10.2016	TS1b	c
171	21.10.2016	TS1b	c
172	21.10.2016	TS1b	c
173	28.10.2016	TS1b	f
174	28.10.2016	TS1b	c
175	28.10.2016	TS1b	c
176	28.10.2016	TS1b	c
177	28.10.2016	TS1b	c
178	28.10.2016	TS1b	c
179	28.10.2016	TS1b	c
180	28.10.2016	TS1b	c
181	28.10.2016	TS1b	c
182	28.10.2016	TS1b	c
183	31.10.2016	TS1b	c
184	31.10.2016	TS1b	f
185	31.10.2016	TS1b	c
186	31.10.2016	TS1b	c
187	31.10.2016	TS1b	c
188	31.10.2016	TS1b	c
189	31.10.2016	TS1b	c
190	31.10.2016	TS1b	c
191	31.10.2016	TS1b	c
192	31.10.2016	TS1b	c
193	31.10.2016	TS1b	c
194	31.10.2016	TS1b	c
195	31.10.2016	TS1b	f
196	31.10.2016	TS1b	c
197	31.10.2016	TS1b	c
198	01.11.2016	TS1c	f
199	01.11.2016	TS1c	c
200	01.11.2016	TS1c	c
201	01.11.2016	TS1c	f
202	01.11.2016	TS1c	c
203	01.11.2016	TS1c	c
204	01.11.2016	TS1c	c
205	01.11.2016	TS1c	c
206	01.11.2016	TS1c	c
207	01.11.2016	TS1c	c

208	01.11.2016	TS1c	c
209	01.11.2016	TS1c	c
210	01.11.2016	TS1c	c
211	01.11.2016	TS1c	c
212	01.11.2016	TS1c	c
213	02.11.2016	TS1c	c
214	02.11.2016	TS1c	c
215	02.11.2016	TS1c	c
216	02.11.2016	TS1c	f
217	02.11.2016	TS1c	c
218	02.11.2016	TS1c	c
219	02.11.2016	TS1c	c
220	02.11.2016	TS1c	c
221	02.11.2016	TS1c	f
222	02.11.2016	TS1c	c
223	02.11.2016	TS1c	c
224	02.11.2016	TS1c	c
225	02.11.2016	TS1c	c
226	02.11.2016	TS1c	c
227	02.11.2016	TS1c	c
228	03.11.2016	TS1c	c
229	03.11.2016	TS1c	c
230	03.11.2016	TS1c	f
231	03.11.2016	TS1c	c
232	03.11.2016	TS1c	c
233	03.11.2016	TS1c	c
234	03.11.2016	TS1c	c
235	03.11.2016	TS1c	c
236	03.11.2016	TS1c	c
237	03.11.2016	TS1c	c
238	03.11.2016	TS1c	c
239	03.11.2016	TS1c	c
240	03.11.2016	TS1c	c
241	03.11.2016	TS1c	c
242	03.11.2016	TS1c	c
243	04.11.2016	TS2	f
244	04.11.2016	TS2	c
245	04.11.2016	TS2	c
246	04.11.2016	TS2	f
247	04.11.2016	TS2	c
248	04.11.2016	TS2	c
249	04.11.2016	TS2	c
250	04.11.2016	TS2	c
251	04.11.2016	TS2	c
252	04.11.2016	TS2	f

253	04.11.2016	TS2	f
254	04.11.2016	TS2	f
255	04.11.2016	TS2	f
256	04.11.2016	TS2	c
257	04.11.2016	TS2	c
258	07.11.2016	TS2	c
259	07.11.2016	TS2	c
260	07.11.2016	TS2	c
261	07.11.2016	TS2	c
262	07.11.2016	TS2	f
263	07.11.2016	TS2	f
264	07.11.2016	TS2	c
265	08.11.2016	TS2	c
266	08.11.2016	TS2	f
267	08.11.2016	TS2	f
268	08.11.2016	TS2	f
269	08.11.2016	TS2	f
270	08.11.2016	TS2	f
271	08.11.2016	TS2	c
272	08.11.2016	TS2	c
273	08.11.2016	TS2	c
274	08.11.2016	TS2	c
275	08.11.2016	TS2	c
276	08.11.2016	TS2	f
277	09.11.2016	TS2	c
278	09.11.2016	TS2	c
279	09.11.2016	TS2	c
280	09.11.2016	TS2	c
281	09.11.2016	TS2	c
282	09.11.2016	TS2	c
283	09.11.2016	TS2	c
284	09.11.2016	TS2	c
285	09.11.2016	TS2	c
286	09.11.2016	TS2	c
287	09.11.2016	TS2	c
288	09.11.2016	TS2	c
289	09.11.2016	TS2	c
290	09.11.2016	TS2	c
291	09.11.2016	TS2	c
292	10.11.2016	TS2	c
293	10.11.2016	TS2	c
294	10.11.2016	TS2	c
295	10.11.2016	TS2	f
296	10.11.2016	TS2	c
297	10.11.2016	TS2	c

298	10.11.2016	TS2	c
299	10.11.2016	TS2	c
300	10.11.2016	TS2	c
301	10.11.2016	TS2	c
302	10.11.2016	TS2	c
303	10.11.2016	TS2	c
304	10.11.2016	TS2	c
305	10.11.2016	TS2	c
306	10.11.2016	TS2	f
307	11.11.2016	TS3	f
308	11.11.2016	TS3	c
309	11.11.2016	TS3	f
310	11.11.2016	TS3	c
311	11.11.2016	TS3	f
312	11.11.2016	TS3	c
313	11.11.2016	TS3	c
314	11.11.2016	TS3	c
315	11.11.2016	TS3	c
316	11.11.2016	TS3	c
317	11.11.2016	TS3	c
318	11.11.2016	TS3	f
319	11.11.2016	TS3	c
320	11.11.2016	TS3	c
321	11.11.2016	TS3	f
322	14.11.2016	TS3	f
323	14.11.2016	TS3	f
324	14.11.2016	TS3	c
325	14.11.2016	TS3	c
326	14.11.2016	TS3	c
327	14.11.2016	TS3	c
328	14.11.2016	TS3	f
329	14.11.2016	TS3	c
330	14.11.2016	TS3	c
331	14.11.2016	TS3	c
332	15.11.2016	TS3	c
333	15.11.2016	TS3	c
334	15.11.2016	TS3	c
335	15.11.2016	TS3	c
336	15.11.2016	TS3	c
337	15.11.2016	TS3	c
338	15.11.2016	TS3	f
339	15.11.2016	TS3	c
340	15.11.2016	TS3	c
341	15.11.2016	TS3	c
342	15.11.2016	TS3	c

343	15.11.2016	TS3	c
344	15.11.2016	TS3	c
345	15.11.2016	TS3	c
346	15.11.2016	TS3	c

Table S7: Raw data of LIL in October-November 2016. A "c" stands for choosing the rewarded corridor (= correct). An "f" stands for choosing the non-rewarded corridor (= false).

**Trial Number**	**Date**	**Test Series**	**correct/false**
1	04.10.2016	TS1a	f
2	04.10.2016	TS1a	c
3	04.10.2016	TS1a	f
4	04.10.2016	TS1a	c
5	04.10.2016	TS1a	f
6	04.10.2016	TS1a	c
7	04.10.2016	TS1a	f
8	04.10.2016	TS1a	c
9	04.10.2016	TS1a	c
10	04.10.2016	TS1a	c
11	05.10.2016	TS1a	f
12	05.10.2016	TS1a	c
13	05.10.2016	TS1a	c
14	05.10.2016	TS1a	f
15	05.10.2016	TS1a	c
16	05.10.2016	TS1a	c
17	05.10.2016	TS1a	c
18	05.10.2016	TS1a	f
19	05.10.2016	TS1a	c
20	05.10.2016	TS1a	c
21	06.10.2016	TS1a	c
22	06.10.2016	TS1a	f
23	06.10.2016	TS1a	c
24	06.10.2016	TS1a	c
25	06.10.2016	TS1a	c
26	06.10.2016	TS1a	f
27	06.10.2016	TS1a	f
28	06.10.2016	TS1a	c
29	06.10.2016	TS1a	c
30	06.10.2016	TS1a	c
31	06.10.2016	TS1a	c
32	06.10.2016	TS1a	c
33	06.10.2016	TS1a	f

34	06.10.2016	TS1a	c
35	06.10.2016	TS1a	f
36	07.10.2016	TS1a	f
37	07.10.2016	TS1a	c
38	07.10.2016	TS1a	c
39	07.10.2016	TS1a	c
40	07.10.2016	TS1a	f
41	07.10.2016	TS1a	f
42	07.10.2016	TS1a	f
43	07.10.2016	TS1a	c
44	07.10.2016	TS1a	c
45	07.10.2016	TS1a	c
46	10.10.2016	TS1a	f
47	10.10.2016	TS1a	c
48	10.10.2016	TS1a	c
49	10.10.2016	TS1a	c
50	10.10.2016	TS1a	f
51	10.10.2016	TS1a	c
52	10.10.2016	TS1a	f
53	10.10.2016	TS1a	c
54	10.10.2016	TS1a	f
55	10.10.2016	TS1a	c
56	11.10.2016	TS1a	f
57	11.10.2016	TS1a	c
58	11.10.2016	TS1a	c
59	11.10.2016	TS1a	c
60	11.10.2016	TS1a	c
61	11.10.2016	TS1a	c
62	11.10.2016	TS1a	f
63	11.10.2016	TS1a	c
64	11.10.2016	TS1a	c
65	11.10.2016	TS1a	f
66	11.10.2016	TS1a	f
67	11.10.2016	TS1a	c
68	11.10.2016	TS1a	c
69	11.10.2016	TS1a	f
70	11.10.2016	TS1a	f
71	12.10.2016	TS1a	f
72	12.10.2016	TS1a	c
73	12.10.2016	TS1a	f
74	12.10.2016	TS1a	c
75	12.10.2016	TS1a	f
76	12.10.2016	TS1a	c
77	12.10.2016	TS1a	c
78	12.10.2016	TS1a	c

79	12.10.2016	TS1a	c
80	12.10.2016	TS1a	c
81	12.10.2016	TS1a	c
82	12.10.2016	TS1a	c
83	12.10.2016	TS1a	f
84	12.10.2016	TS1a	c
85	12.10.2016	TS1a	c
86	13.10.2016	TS1a	f
87	13.10.2016	TS1a	c
88	13.10.2016	TS1a	c
89	13.10.2016	TS1a	c
90	13.10.2016	TS1a	c
91	13.10.2016	TS1a	c
92	13.10.2016	TS1a	c
93	13.10.2016	TS1a	c
94	13.10.2016	TS1a	c
95	13.10.2016	TS1a	f
96	14.10.2016	TS1a	c
97	14.10.2016	TS1a	f
98	14.10.2016	TS1a	c
99	14.10.2016	TS1a	c
100	14.10.2016	TS1a	c
101	14.10.2016	TS1a	c
102	14.10.2016	TS1a	c
103	14.10.2016	TS1a	c
104	14.10.2016	TS1a	c
105	14.10.2016	TS1a	f
106	14.10.2016	TS1a	f
107	14.10.2016	TS1a	c
108	14.10.2016	TS1a	c
109	14.10.2016	TS1a	f
110	17.10.2016	TS1a	f
111	17.10.2016	TS1a	f
112	17.10.2016	TS1a	f
113	17.10.2016	TS1a	c
114	17.10.2016	TS1a	c
115	17.10.2016	TS1a	c
116	17.10.2016	TS1a	c
117	17.10.2016	TS1a	c
118	17.10.2016	TS1a	c
119	17.10.2016	TS1a	c
120	18.10.2016	TS1a	c
121	18.10.2016	TS1a	f
122	18.10.2016	TS1a	c
123	18.10.2016	TS1a	c

124	18.10.2016	TS1a	c
125	18.10.2016	TS1a	c
126	18.10.2016	TS1a	c
127	18.10.2016	TS1a	c
128	18.10.2016	TS1a	c
129	18.10.2016	TS1a	c
130	19.10.2016	TS1b	c
131	19.10.2016	TS1b	c
132	19.10.2016	TS1b	f
133	19.10.2016	TS1b	c
134	19.10.2016	TS1b	c
135	19.10.2016	TS1b	c
136	19.10.2016	TS1b	c
137	19.10.2016	TS1b	c
138	19.10.2016	TS1b	f
139	21.10.2016	TS1b	c
140	21.10.2016	TS1b	c
141	21.10.2016	TS1b	c
142	21.10.2016	TS1b	c
143	21.10.2016	TS1b	c
144	21.10.2016	TS1b	c
145	21.10.2016	TS1b	c
146	21.10.2016	TS1b	f
147	21.10.2016	TS1b	f
148	21.10.2016	TS1b	c
149	21.10.2016	TS1b	c
150	26.10.2016	TS1c	f
151	26.10.2016	TS1c	c
152	26.10.2016	TS1c	f
153	26.10.2016	TS1c	c
154	26.10.2016	TS1c	c
155	26.10.2016	TS1c	c
156	26.10.2016	TS1c	c
157	26.10.2016	TS1c	f
158	26.10.2016	TS1c	f
159	26.10.2016	TS1c	c
160	26.10.2016	TS1c	c
161	26.10.2016	TS1c	c
162	28.10.2016	TS1c	f
163	28.10.2016	TS1c	c
164	28.10.2016	TS1c	f
165	28.10.2016	TS1c	c
166	28.10.2016	TS1c	f
167	28.10.2016	TS1c	c
168	28.10.2016	TS1c	c

169	28.10.2016	TS1c	c
170	28.10.2016	TS1c	c
171	28.10.2016	TS1c	c
172	28.10.2016	TS1c	c
173	28.10.2016	TS1c	f
174	31.10.2016	TS1c	c
175	31.10.2016	TS1c	c
176	31.10.2016	TS1c	c
177	31.10.2016	TS1c	c
178	31.10.2016	TS1c	f
179	31.10.2016	TS1c	c
180	01.11.2016	TS1c	c
181	01.11.2016	TS1c	c
182	01.11.2016	TS1c	f
183	01.11.2016	TS1c	c
184	01.11.2016	TS1c	f
185	01.11.2016	TS1c	c
186	01.11.2016	TS1c	f
187	01.11.2016	TS1c	c
188	01.11.2016	TS1c	c
189	01.11.2016	TS1c	c
190	01.11.2016	TS1c	c
191	01.11.2016	TS1c	c
192	01.11.2016	TS1c	c
193	01.11.2016	TS1c	f
194	01.11.2016	TS1c	c
195	02.11.2016	TS1c	f
196	02.11.2016	TS1c	f
197	02.11.2016	TS1c	f
198	02.11.2016	TS1c	c
199	02.11.2016	TS1c	c
200	02.11.2016	TS1c	c
201	02.11.2016	TS1c	f
202	02.11.2016	TS1c	c
203	02.11.2016	TS1c	c
204	02.11.2016	TS1c	c
205	02.11.2016	TS1c	c
206	02.11.2016	TS1c	c
207	02.11.2016	TS1c	f
208	02.11.2016	TS1c	f
209	04.11.2016	TS1c	c
210	04.11.2016	TS1c	c
211	04.11.2016	TS1c	c
212	04.11.2016	TS1c	c
213	04.11.2016	TS1c	c

214	04.11.2016	TS1c	c
215	04.11.2016	TS1c	c
216	04.11.2016	TS1c	c
217	07.11.2016	TS1c	f
218	07.11.2016	TS1c	f
219	07.11.2016	TS1c	c
220	07.11.2016	TS1c	f
221	07.11.2016	TS1c	f
222	07.11.2016	TS1c	c
223	07.11.2016	TS1c	c
224	07.11.2016	TS1c	c
225	07.11.2016	TS1c	c
226	07.11.2016	TS1c	c
227	07.11.2016	TS1c	c
228	07.11.2016	TS1c	c
229	07.11.2016	TS1c	c
230	07.11.2016	TS1c	c
231	07.11.2016	TS1c	c
232	08.11.2016	TS1c	f
233	08.11.2016	TS1c	f
234	08.11.2016	TS1c	f
235	08.11.2016	TS1c	c
236	08.11.2016	TS1c	c
237	08.11.2016	TS1c	c
238	08.11.2016	TS1c	c
239	08.11.2016	TS1c	c
240	08.11.2016	TS1c	c
241	08.11.2016	TS1c	c
242	08.11.2016	TS1c	c
243	08.11.2016	TS1c	c
244	08.11.2016	TS1c	c
245	09.11.2016	TS1c	c
246	09.11.2016	TS1c	c
247	09.11.2016	TS1c	c
248	09.11.2016	TS1c	c
249	09.11.2016	TS1c	c
250	09.11.2016	TS1c	c
251	09.11.2016	TS1c	c
252	09.11.2016	TS1c	c
253	09.11.2016	TS1c	c
254	09.11.2016	TS1c	c
255	11.11.2016	TS2	f
256	11.11.2016	TS2	f
257	11.11.2016	TS2	f
258	11.11.2016	TS2	c

259	11.11.2016	TS2	f
260	11.11.2016	TS2	c
261	11.11.2016	TS2	f
262	11.11.2016	TS2	c
263	11.11.2016	TS2	c
264	11.11.2016	TS2	c
265	11.11.2016	TS2	c
266	11.11.2016	TS2	f
267	11.11.2016	TS2	c
268	11.11.2016	TS2	c
269	11.11.2016	TS2	c
270	14.11.2016	TS2	c
271	14.11.2016	TS2	c
272	14.11.2016	TS2	c
273	14.11.2016	TS2	c
274	14.11.2016	TS2	c
275	14.11.2016	TS2	c
276	14.11.2016	TS2	c
277	14.11.2016	TS2	c
278	14.11.2016	TS2	f
279	14.11.2016	TS2	c
280	14.11.2016	TS2	f
281	14.11.2016	TS2	c
282	14.11.2016	TS2	c
283	14.11.2016	TS2	c
284	14.11.2016	TS2	c
285	15.11.2016	TS2	f
286	15.11.2016	TS2	c
287	15.11.2016	TS2	f
288	15.11.2016	TS2	c
289	15.11.2016	TS2	c
290	15.11.2016	TS2	c
291	16.11.2016	TS2	c
292	16.11.2016	TS2	c
293	16.11.2016	TS2	c
294	16.11.2016	TS2	c
295	16.11.2016	TS2	c
296	16.11.2016	TS2	c
297	16.11.2016	TS2	c
298	16.11.2016	TS2	c
299	16.11.2016	TS2	c
300	16.11.2016	TS2	c
301	16.11.2016	TS2	c
302	16.11.2016	TS2	f
303	21.11.2016	TS3	c

304	21.11.2016	TS3	c
305	21.11.2016	TS3	c
306	21.11.2016	TS3	f
307	21.11.2016	TS3	c
308	21.11.2016	TS3	c
309	21.11.2016	TS3	f
310	21.11.2016	TS3	c
311	21.11.2016	TS3	f
312	21.11.2016	TS3	c
313	21.11.2016	TS3	f
314	21.11.2016	TS3	c
315	21.11.2016	TS3	c
316	22.11.2016	TS3	c
317	22.11.2016	TS3	c
318	22.11.2016	TS3	c
319	22.11.2016	TS3	c
320	22.11.2016	TS3	c
321	22.11.2016	TS3	c
322	22.11.2016	TS3	f
323	22.11.2016	TS3	c
324	22.11.2016	TS3	c
325	22.11.2016	TS3	f
326	23.11.2016	TS3	c
327	23.11.2016	TS3	f
328	23.11.2016	TS3	f
329	23.11.2016	TS3	c
330	23.11.2016	TS3	c
331	23.11.2016	TS3	c
332	23.11.2016	TS3	c
333	23.11.2016	TS3	f
334	23.11.2016	TS3	c
335	23.11.2016	TS3	c
336	23.11.2016	TS3	c
